# Technology-driven approaches to intelligent mechanical weed control: a systematic review for sustainable weed management

**DOI:** 10.3389/fpls.2025.1734507

**Published:** 2025-12-18

**Authors:** Samriddha Das, Arjun Upadhyay, Xin Sun

**Affiliations:** Department of Agricultural and Biosystems Engineering, North Dakota State University, Fargo, ND, United States

**Keywords:** artificial intelligence, automated weeding systems, machine vision, precision agriculture, robotic weed control

## Abstract

The intensifying global demand for sustainable agriculture has necessitated innovation in weed management, particularly through intelligent, non-chemical alternatives. Among these, smart mechanical weeding systems integrating artificial intelligence (AI), machine vision, and robotics are emerging as transformative tools for precise and eco-friendly weed control. While several recent reviews have examined intelligent weeding or machine vision-based weed management more broadly, a comprehensive and systematically structured synthesis focusing specifically on AI-driven mechanical weeding systems that integrate both vision and robotic actuation remains limited. This study presents a systematic review of 176 technical papers published between 2000 and 2024, with in-depth analysis of 33 key works, aiming to explore the design and performance of intelligent mechanical weed control systems in precision agriculture. The review investigates foundational mechanical weeding methods, recent advances in sensor integration and weed detection algorithms, and the use of robotic platforms for intra- and inter-row weeding. It highlights the critical role of RGB, LiDAR, hyperspectral sensors, and deep learning models in enabling real-time, selective weed removal. Comparative case studies showcase end effectors, control architecture, sensors, and techniques involved across diverse platforms. While significant progress has been made, challenges persist in weed-crop differentiation, model generalization, real-time actuation, and economic feasibility. The review proposes a set of design and operational guidelines addressing sensor fusion, adaptive tooling, platform modularity, and user-centric interfaces. This work provides a targeted, system-level roadmap for researchers, developers, and stakeholders in agricultural robotics, offering insights into current capabilities, gaps, and future directions to advance intelligent mechanical weeding for scalable and sustainable food production.

## 1 Introduction

Weeding is the process of removing unnecessary plants from farming and agricultural lands to prevent them from competing with desired ones over natural resources. These plants are generally recognized by their unpredictable growing locations along with their ability to reproductively proliferate without human intervention (Gao and Su, 2024). The existence of weeds adversely affects crop yield and leads to irregular maturation of crops, thereby complicating the harvesting process due to their disruption of consistent crop growth (Tshewang et al., 2016; Yu et al., 2019). For instance, weeds result in considerable yield losses in wheat, with winter wheat losses in the United States averaging 25.6%. This, combined with a 23.4% loss in Canada, culminates in a potential annual economic deficit of approximately $2.19 billion. Furthermore, for spring wheat, the losses average 33.2% in the United States and 19.5% when combined with Canada, incurring costs up to $1.39 billion. This situation emphasizes the escalating threat posed by weeds (Flessner et al., 2021). Effective weed management is imperative to alleviate the challenges presented by these plants and to enhance crop productivity while sustaining agricultural profitability. Although these practices are labor-intensive and costly, often necessitating substantial manpower and resources, there are instances where the expenses related to weed management may exceed the economic benefits derived from crop production. This highlights the necessity to assess traditional weed management practices and their inherent limitations (Dhakal et al., 2024).

The selection of weeding practices and implements is influenced by factors such as crop type, soil characteristics, and field conditions. Hand-weeding is commonly adopted for smaller landholdings, provided there is sufficient labor availability (Abebe, 2024). Broadly, traditional weeding methods can be categorized into three distinct approaches: physical, chemical, and biological. Each approach offers specific limitations, based on their operational efficiency, environmental impact, and cost-effectiveness (Bond et al., 2003; Gao and Su, 2024). Physical weeding techniques involve thermal technologies, such as laser and flame weeding, effectively controlling weeds but also come up with several challenges. The high temperatures generated in these processes can ignite dry materials in the field, creating fire hazards. Additionally, these methods can pose risks to humans and animals nearby. Flame weeding, in particular, significantly contributes to greenhouse gas emissions, raising environmental concerns due to its detrimental ecological impact (Sivesind et al., 2009). Mechanical weeding is another physical weeding technique and is a widely used traditional practice, with reported average weed removal efficiencies of approximately 80% based on reductions in both weed density and biomass (Jabran and Chauhan, 2015; Liu et al., 2023). However, it also has certain drawbacks. Tools like chain harrows, used for inter-row weeding, can physically damage crops, leading to bruising and stem breakage, which may inhibit plant growth and increase vulnerability to pathogen infestations. Moreover, the heavy design of mechanical weeding equipment can cause soil compaction, adversely affecting soil aeration and root development. Biological weed control employs living organisms, such as insects, fungi, or bacteria, or their byproducts, to suppress weed populations and lessen their impact on crops. Although this method aligns well with environmental principles, its practical application is limited by slow activation and response times, alongside the short half-life of these biological agents. Additionally, the use of bioherbicides derived from these organisms poses potential risks to human and animal health and may have unintended environmental repercussions, thus limiting their widespread adoption in sustainable agricultural systems. Chemical weed control remains the most effective and widely used method for managing weeds, primarily due to the effectiveness of herbicides against various weed populations. However, its extensive application brings significant drawbacks. Chemical herbicides can be quite expensive, increasing the financial burden on agricultural production. Prolonged exposure to these chemicals presents serious health risks to both humans and animals. For example, glyphosate, a widely used herbicide, was classified as “probably carcinogenic to humans” by the World Health Organization in 2015 (Van Bruggen et al., 2018). Furthermore, the significant use of chemical herbicides leads to environmental pollution, including soil contamination, water runoff, and harm to non-target organisms, raising concerns about their sustainability in agricultural practices.

To overcome the limitations of conventional weeding methods, intelligent weeding technologies have emerged as a key component of modern precision agriculture. Unlike traditional Integrated Weed Management (IWM), which combines general mechanical and chemical strategies, intelligent weeding integrates automation, sensing and artificial intelligence to achieve targeted, data-driven weed control (Riemens et al., 2022). Depending on the mode of actuation, intelligent weeding can be categorized into intelligent chemical, intelligent physical and intelligent mechanical approaches (Gerhards et al., 2022; Jiao et al., 2024). Intelligent chemical weeding employs site-specific or variable rate herbicide applications guided by machine vision, thereby reducing chemical usage (Upadhyay et al., 2024a). Intelligent physical weeding utilizes non-chemical energy sources – such as lasers or thermal radiation – to destroy weeds relying on imaging technologies (Bajwa et al., 2015; T. Jin and Han, 2024; Li et al., 2022). Meanwhile intelligent mechanical weeding physically removes or disrupts weeds using robotic end effectors and machine vision, offering an eco-friendly and residue free alternative (Jiang et al., 2023b; Visentin et al., 2023). Collectively, these intelligent systems align with sustainability and resource optimization objectives by minimizing chemical inputs, improving accuracy, and reducing labor requirements.

Despite the benefits of intelligent weed management, the escalating occurrence of herbicide resistant weeds has intensified the need for sustainable, non-chemical control strategies (Délye et al., 2013). Although technologies such as variable-rate spraying, site specific delivery and see and spray systems have improved herbicide efficiency, they can inadvertently accelerate the evolution of resistant weed biotypes (Chang et al., 2023; Upadhyay et al., 2024a). This growing resistance combined with the limited pipeline of new herbicides, underscores the urgency of adopting other non-chemical solutions (Nath et al., 2024). In this context, intelligent mechanical weeding stands out as a promising direction, integrating the precision of robotics with the selectivity of machine vision to address both inter- and intra-row weeds effectively. As a non-chemical method, it not only mitigates herbicide resistance, but also reduces soil and water contamination, fuel consumption and overall environmental burden (Machleb et al., 2020).

Mechanical weed control involves the physical removal or destruction of weeds through direct interaction with the soil and vegetation. The primary techniques employed in mechanical weeding include tillage, cutting, and pulling (Cloutier et al., 2007; Hussain et al., 2018; McCool et al., 2018). These methods disrupt weed growth by turning the soil and uprooting root systems thereby inflicting lethal injuries that prevent regrowth (Zawada et al., 2023). Their effectiveness largely depends on the method, timing, and intensity of operation (Machleb et al., 2020). Despite their effectiveness, conventional mechanical weeding approaches often lead to crop damage followed by soil compaction, adversely affecting soil aeration and root development (Pannacci et al., 2017). Moreover, the performance of mechanical weeders is influenced by field conditions and weather variability, which limit their adaptability across diverse agricultural contexts. Additional issues such as higher energy, fuel consumption and labor demand also reduce their economic feasibility (Hussain et al., 2018).

Recent advances have integrated automation, robotics, and computer vision into mechanical weeding, giving rise to intelligent mechanical weeders. These systems employ sensors and AI algorithms for real-time weed detection and selective removal, minimizing crop disturbance and operator dependency. Specialized end effectors such as finger weeders, rotary cultivators, and elastic comb mechanisms effectively manage coplex intra-row weeds that were difficult to control manually (Chang et al., 2021). For instance, the SMART CULTIVATOR by Stout Industrial Technology, uses True Vision software for crop recognition and adaptive blade control. It handles diverse crops including artichokes, broccoli, cabbage, and pumpkins—with 99% identification accuracy, 1-2 acres/h field coverage, and up to 96% labor reduction compared with manual weeding (Stout, 2020). These systems perform best at early weed-growth stages, roughly two to three weeks after sowing, when weeds are spatially distinct (Jabran and Chauhan, 2015). Under dense weed canopies, their precision declines, favoring hybrid strategies that pair conventional tools (e.g., harrows or hoes) for inter-row control with AI-based weeders for intra-row precision-maximizing efficiency, minimizing environmental impact and advancing sustainable resource optimized agriculture.

This review therefore aims to critically examine the potential and limitations of intelligent mechanical weed control, focusing on its integration with advanced sensing, perception, and actuation systems. It further identifies the technological gaps, operational bottlenecks, and future research pathways required to develop scalable, efficient, and sustainable robotic solutions that align with the broader goals of precision agriculture.

The recent reviews in weed management have predominantly adopted a comprehensive approach, encompassing the entire spectrum of available weed management techniques. These reviews typically provide an overview of various methods, highlighting their advantages, use cases, and technical findings, offering a holistic perspective on weed management challenges and solutions. However, most of these studies are dedicated to exploring the use of ground robots and UAVs for general weed management, often covering a broad range of aspects without delving deeply into specific techniques. Additionally, these reviews frequently separate the discussion of weed removal and weed detection, even though an integrated approach addressing both domains is critical for developing effective, sustainable solutions.

This paper aims to address the existing research gap by focusing on a critical aspect of weed management—advancements and emerging trends in mechanical weed removal techniques for precise and intelligent weed removal. It demonstrates the use of imaging technologies for accurately localizing and positioning weeds, a foundational component of intelligent mechanical weeding systems. Furthermore, the review incorporates detailed case studies that illustrate prevailing trends and methodologies, along with a comparative analysis of the performance of platforms employing these technologies. By adopting this focused approach, the paper emphasizes the potential of integrating weed detection and removal into a cohesive system, offering valuable insights into contemporary precision agriculture practices.

## 2 Methodology

In the context of a literature review, two primary approaches are commonly employed: systematic review and narrative review. The systematic approach follows a structured methodology that addresses specific technical questions by systematically analyzing relevant research papers. It provides a comprehensive synthesis of results, methodologies, and key findings from existing studies. Conversely, the narrative approach focuses on providing a theoretical background and conceptual understanding of the subject matter, primarily adopting a qualitative perspective rather than a quantitative one (Rother, 2007). This review adopted a systematic approach guided by the PRISMA 2020 guidelines to ensure transparency, reproducibility, and methodological rigor (Page et al., 2021). This approach was chosen over a narrative approach to enable structured identification, screening, and synthesis of peer-reviewed studies addressing both traditional and smart mechanical weed control systems.

**Figure 1:**
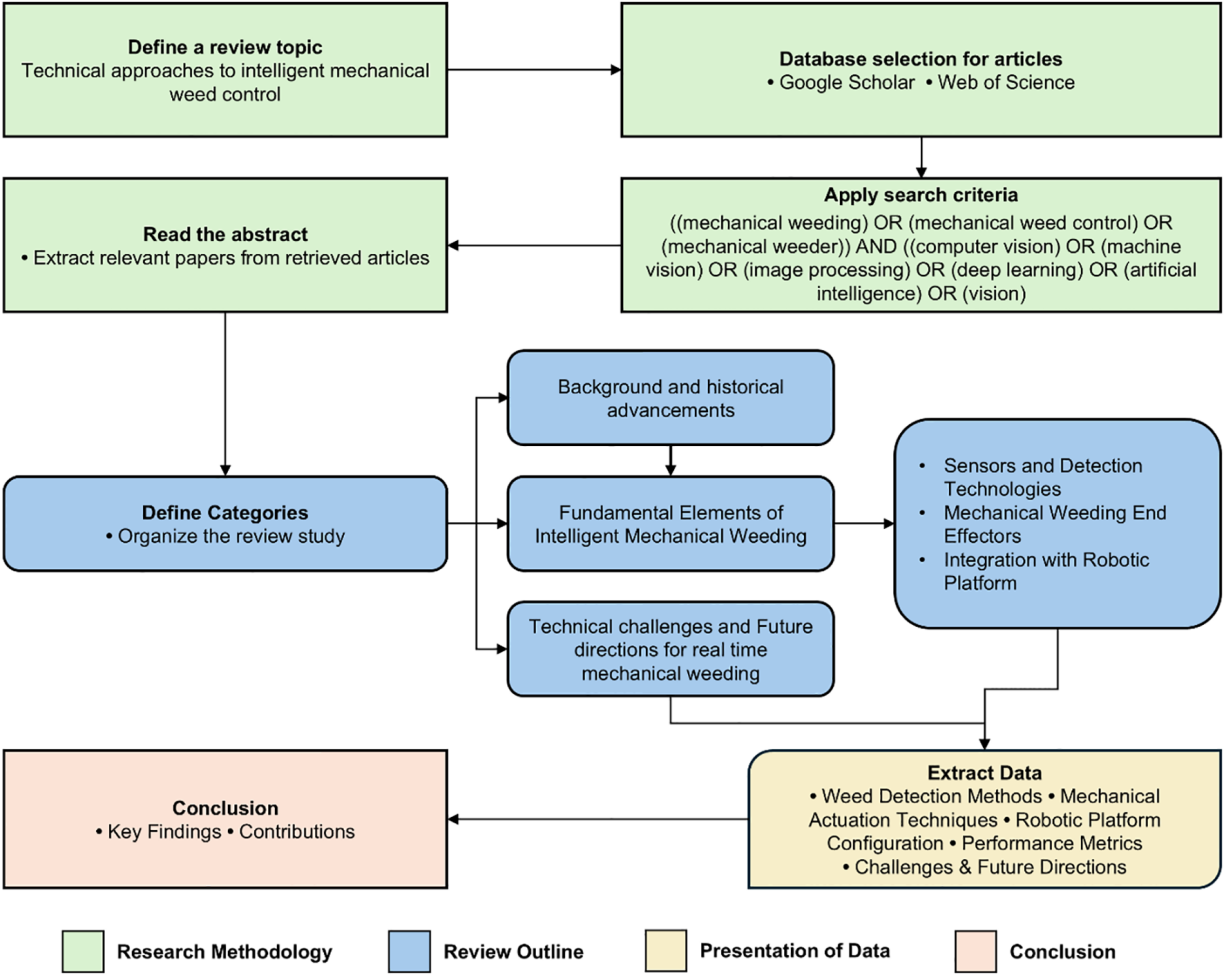
Review paper flowchart layout.

The overall workflow of the review from topic definition through article extraction and synthesis is summarized in **Figure 1**. To retrieve relevant literature, two primary databases-Google Scholar and Web of Science were used between October 2024 to January 2025. These databases were selected for their broad interdisciplinary coverage spanning precision agriculture, agricultural robotics, and artificial intelligence. Cross verification confirmed that most relevant studies, indexed in other major engineering/computer-science databases, were already represented within these two databases ensuring comprehensive inclusion while minimizing redundancy.

A Boolean keyword-based strategy was adopted to capture the widest possible scope of research activity: (“mechanical weeding” OR “mechanical weed control” OR “mechanical weeder”) AND (“computer vision” OR “machine vision” OR “image processing” OR “deep learning” OR “artificial intelligence” OR “vision”).

The initial research retrieved 11,275 publications (Google Scholar = 7430; Web of Science = 3845). Duplicate records were removed through automatic deduplication followed by manual verification yielding 9862 unique articles. Titles and abstracts were screened according to predefined inclusion and exclusion criteria (**Table 1**). The process involved multiple iterations to verify relevance.

**Table 1:** Eligibility criteria and selection process for literature review.

Criterion	Considerations/procedures
Eligibility criteria	Peer-reviewed English-language studies 2000-2024 (total records = 11275)
Database search and keywords	Databases: Google Scholar and Web of Science. Search query: (“mechanical weeding” OR “mechanical weed control” OR “mechanical weeder”) AND (“computer vision” OR “machine vision” OR “image processing” OR “deep learning” OR “artificial intelligence” OR “vision”).
Exclusion criteria	• Duplicate and non-English papers • Review articles • Economic or non-mechanical weed-control studies • Non-technical reports
Quality assessment	Five-domain rubric (design clarity, sensor/actuator details, data availability, validation method, completion report)
Final dataset analysis	176 eligible studies were retained for trend analysis, out of which 33 quality papers were selected for detailed review and comparison

**Figure 2:**
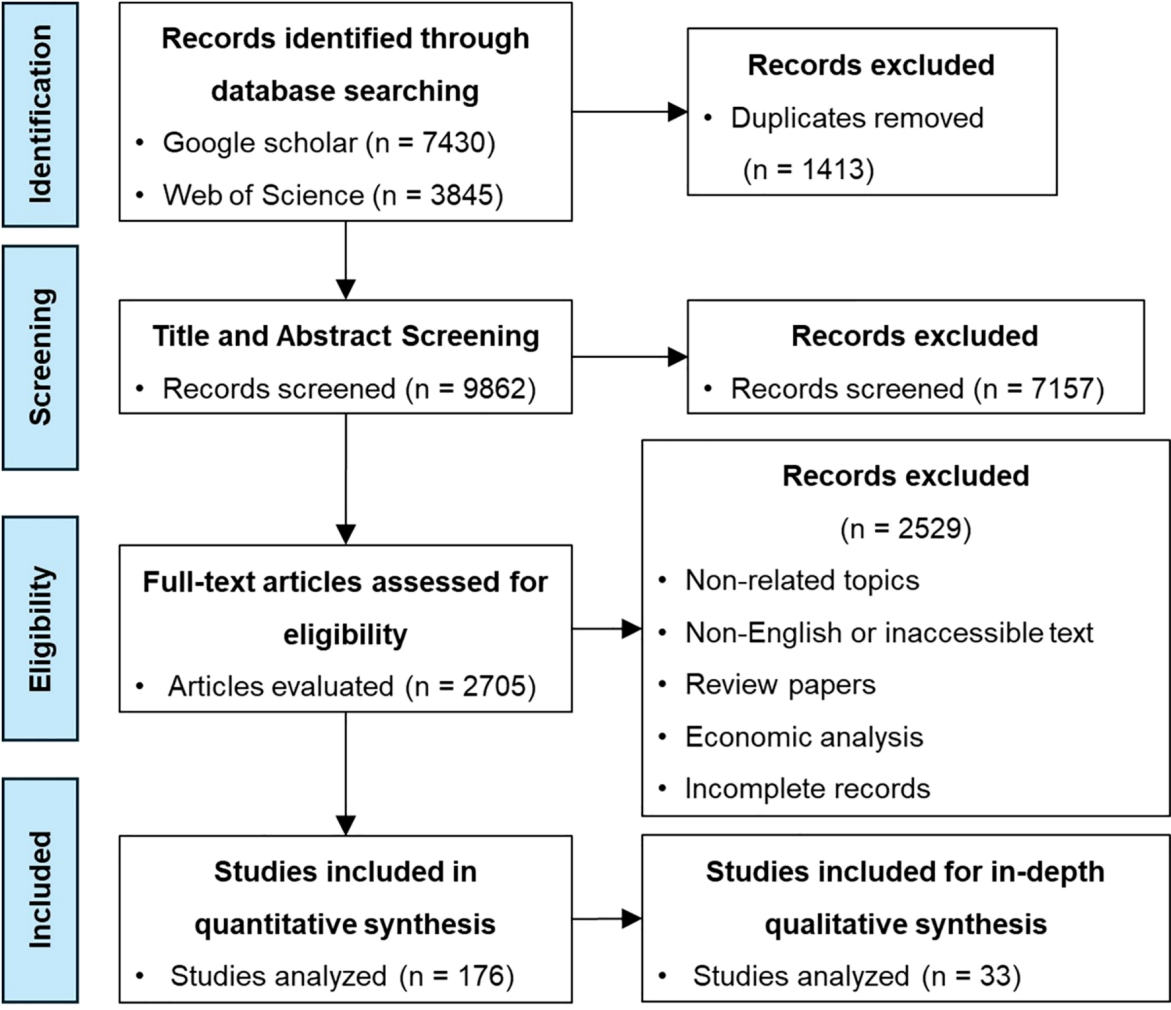
Flow diagram for study selection and inclusion.

After screening, 2705 articles were retained for full-text evaluation. Studies not written in English, review papers, purely economic assessments or those unrelated to mechanical or intelligent mechanical weeding were excluded. Ultimately, 176 technical papers directly addressing mechanical or automated weeding were included for quantitative analysis. From this dataset, 33 focal studies were selected for in-depth synthesis based on quality scoring rubric that assessed five dimensions: (i) design clarity, (ii) details of sensor/actuator integration, (iii) availability of algorithmic or performance data, (iv) validation method and (v) completion report. Each study was rated on a five-point scale (1 = low detail to 5 = comprehensive) with the highest scoring studies forming the analytical subset for further discussion. The complete identification and selection process has been illustrated in the PRISMA flow diagram, **Figure 2**.

**Figure 3:**
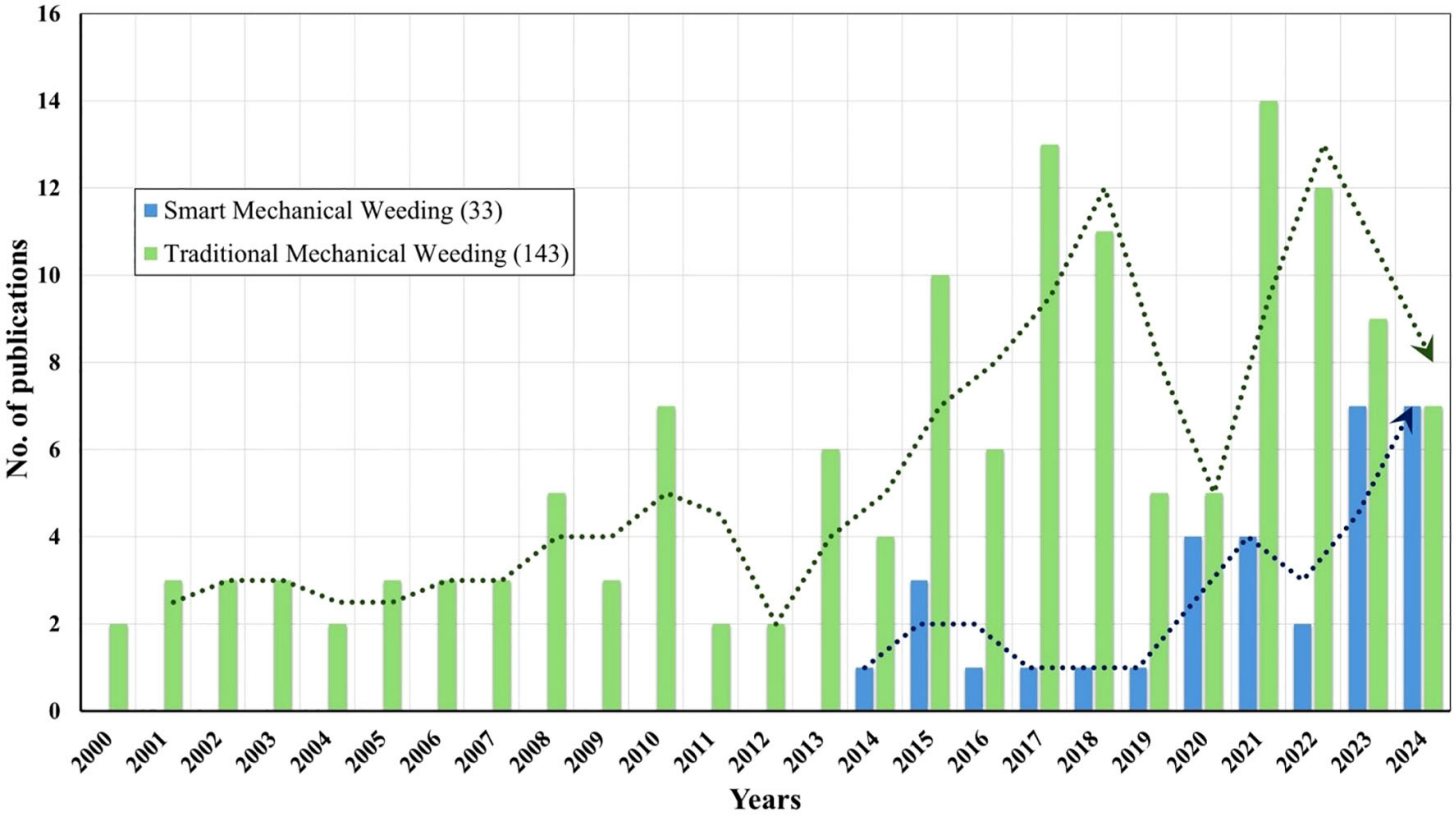
The trend in published peer-reviewed article on mechanical weed control (2000-2024).

Data extracted from the selected papers included mechanical actuation type (rotary, finger, blade, linear, etc.), sensing and detection technology (RGB, LiDAR, multispectral, hyperspectral), (c) robotic actuation configuration and key performance indicators such as precision, accuracy, operational speed etc. Potential sources of bias were qualitatively evaluated by examining the transparency of experimental design, availability of performance data and validation consistency across studies. To contextualize the growth of research in this domain, **Figure 3** illustrates the trend in peer-reviewed publications on traditional and mechanical weeding between 2000 and 2024. The data show an increasing trend in work related to intelligent mechanical weeding after 2019 reflecting the rapid adoption of smart technologies in agricultural automation.

Several previous review studies have investigated different aspects of weed management, including weed control techniques, sensor-based weed detection, robotic weeding systems, and artificial intelligence-driven weed detection approaches (Gao and Su, 2024; Machleb et al., 2020; Rai et al., 2023; Upadhyay et al., 2024b). However, given the breadth of existing literature, the present review adopts a more specialized focus on smart mechanical weeding technologies, emphasizing recent advancements, their integration with imaging sensors for automation and actuation and the technical challenges involved.

## 3 Mechanical weeding: background, evolution, and key components

Mechanical weeding, a practice that employs tools, implements, and machinery for weed control, has been a cornerstone of agricultural weed management since ancient times, complementing manual hand-pulling. This method has demonstrated high effectiveness in eliminating weeds while ensuring no chemical residues are left on crops (Pannacci et al., 2017; Zimdahl, 2018). The mechanical approach primarily involves processes such as cutting, burying, or uprooting weeds, effectively destroying these undesired plants (Hussain et al., 2018).

### 3.1 Background and historical advancements

**Figure 4:**
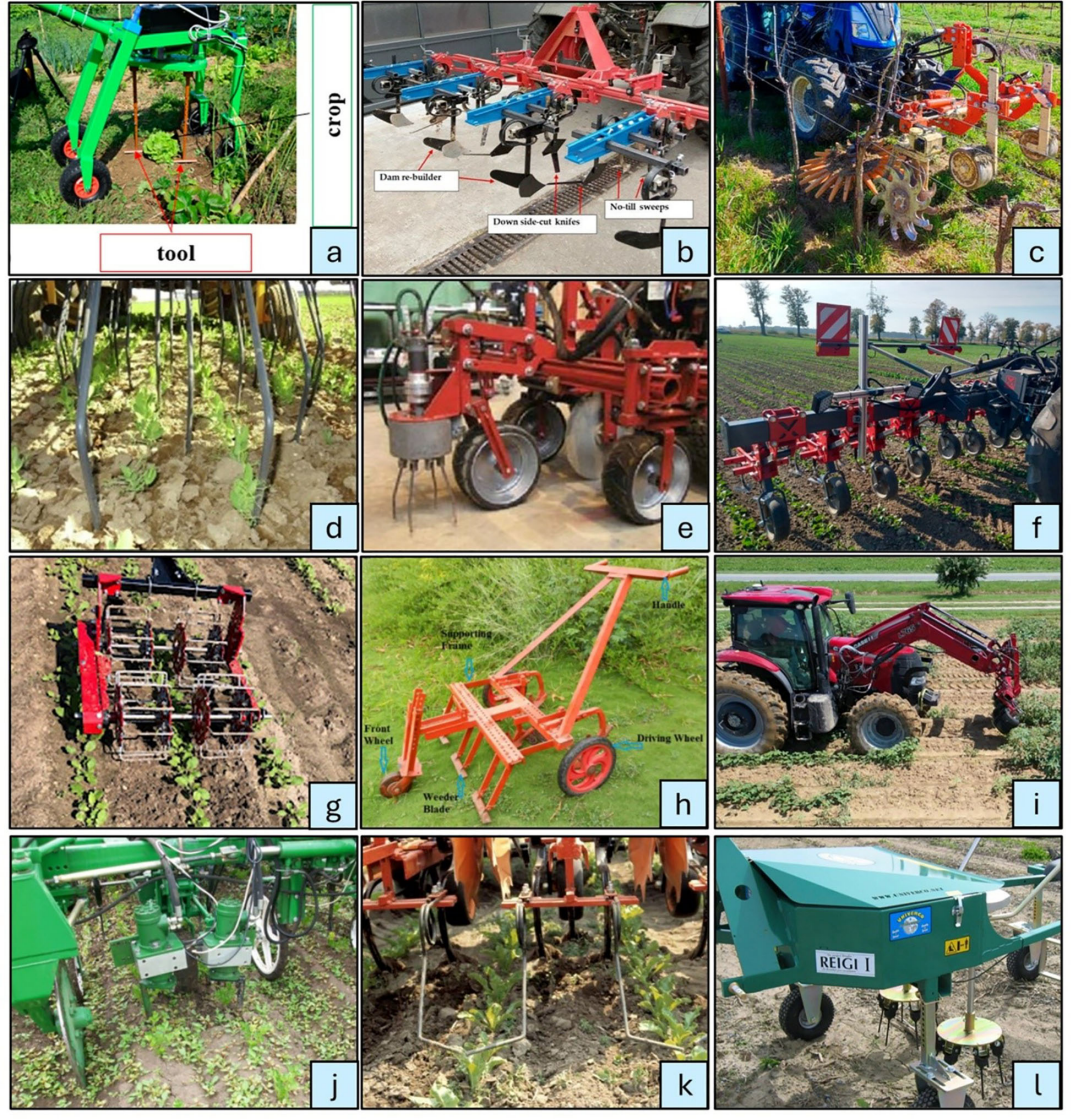
Types of mechanical weeders – **(a)** rotating hoe weeder (Trajkovski et al., 2024), **(b)** inter-row hoe (Alagbo et al., 2022), **(c)** roll hackle and finger weeder (Gagliardi et al., 2023), **(d)** flex tine weeder (Machleb et al., 2020) **(e)** cycloid hoe weeder (Rueda-Ayala et al., 2010), **(f)** sweep type cultivator weeder (Zawada et al., 2023), **(g)** basket weeder (Hoidal, 2019), **(h)** manual push blade weeder (Rajashekar et al., 2014), **(i)** Bourquin Organic Weedpuller (Moore et al., 2023), **(j)** inter-row cultivator weeder (Fennimore et al., 2013), **(k)** torsion weeder (Pannacci et al., 2018), **(l)** vertical tine eco-weeder (Ahmad et al., 2014).

A wide range of mechanical weeding tools and equipment has been utilized over time, including hoes, split-hoes, brush weeders, robotic weeders, row crop cultivators, finger weeders, and tine harrows (Mehdizadeh and Mushtaq, 2020). Among these, certain tools, such as flex tines are designed for manual operation, whereas equipment like harrows, rotary hoes and weeders are typically tractor-mounted or automated systems. **Figure 4** gives a demonstration of the popular and widely used mechanical weeding tools which have been in practice over the years. Although alternative weed management techniques exist, mechanical weeding offers distinct advantages as a non-chemical, environmentally friendly approach that avoids pollution while enhancing soil health by loosening and improving soil fertility (Liu et al., 2023).

Traditionally, mechanical weeding relied on tillage operations using tools such as cultivators and rotavators, often integrated with chemical herbicides to improve weed eradication efficiency (Kouwenhoven et al., 1991). A 4-year study on newly planted pecans demonstrated the benefits of this integrated approach, achieving the highest tree diameter increase of 384% with comprehensive herbicide-based weed control, compared to 224% for mowing and 229% for untreated plots.

**Figure 5:**
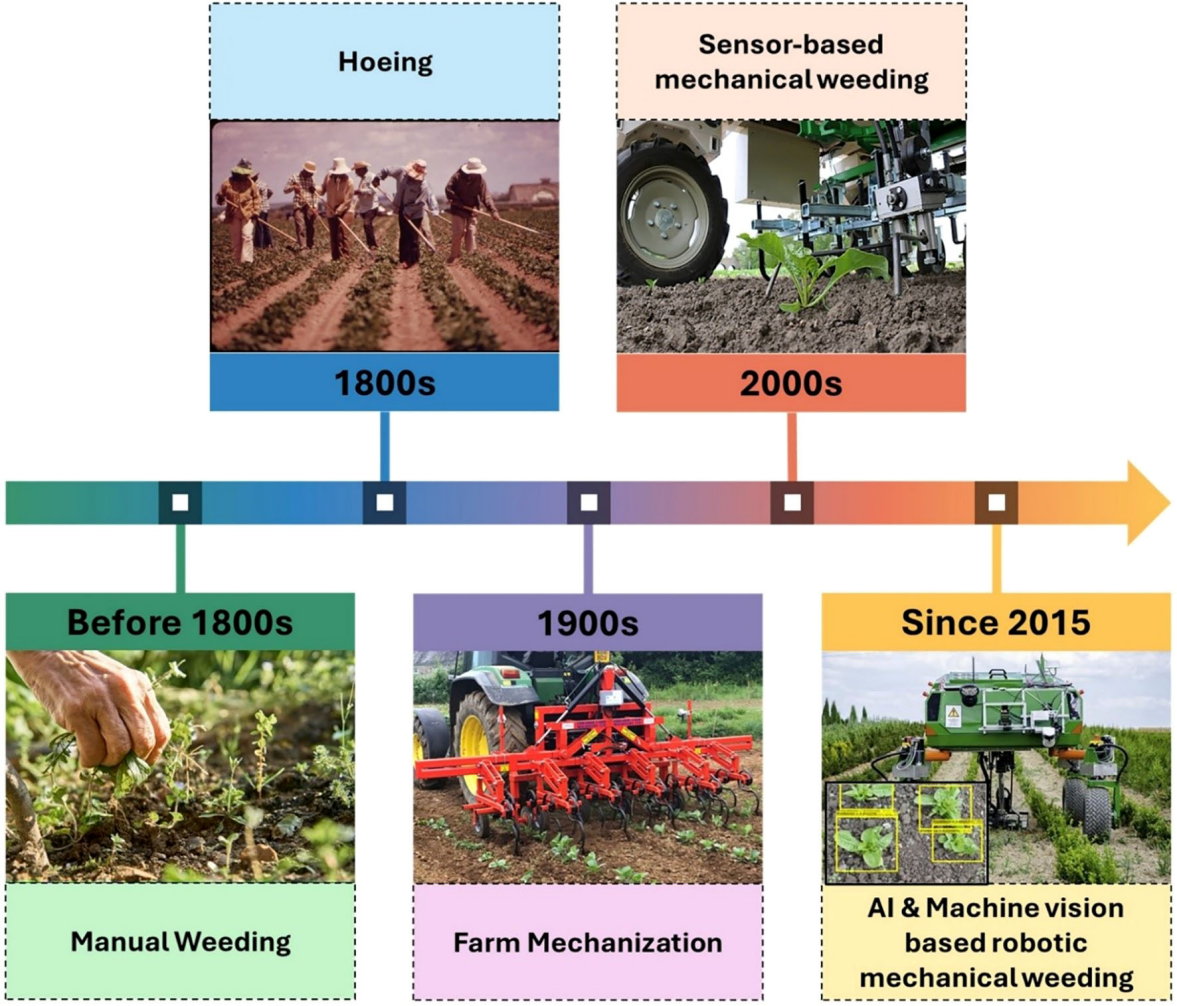
Timeline for advancement in mechanical weeding over the decades.

Disking and selective grass control resulted in 339% and 292% increases, respectively, while irrigation further enhanced cumulative diameter growth to 316% compared to 271% without irrigation (Patterson et al., 1990). However, rising environmental concerns and awareness of the adverse impacts of chemical herbicides have driven a shift towards smart and reduced use of herbicides, leading to the development of advanced machinery and techniques (Parish, 1990). For instance, integrating reduced herbicide rates with interrow cultivation in conservation tillage systems using rotary hoeing for corn effectively controlled weeds and maintained yields comparable to full-rate treatments, providing a sustainable alternative to large scale herbicide usage (Buhler et al., 1995). In another scenario, a mechanical weeder equipped with ground-contoured-following pressing-grass floats (GPF) and weeding rollers, achieved average weeding rates of ~87% in a two-season experiment in a paddy field (Jiao et al., 2022). Field experiments in soybean and sugar beet showed inter row hoeing increased weed control efficacy by 89% in soybean and 87% in sugar beet compared to the conventional methods. Precision hoeing increased the yields by 23 and 28% for sugar beet and soybean respectively (Kunz et al., 2015). **Figure 5** presents a chronological timeline illustrating how mechanical weeding technologies have evolved over the decades—from early manual implements to sophisticated automated and robotic systems—highlighting the progression that has shaped modern intelligent mechanical weeding.

In another experiment, a tractor drawn inter and intra row weeding system was developed for field crops combining active rotary tines for intra row weeding and passive tines for inter row weeding achieving weed mortality of 92.8% in maize and 84.1% in pigeon pea, with plant damage under 6% (Chandel et al., 2021).

The efficacy of mechanical weeding in controlling weed populations and enhancing crop yields, as demonstrated in various studies, underscores its significant role in promoting sustainable agricultural practices. Nevertheless, despite its numerous advantages, mechanical weeding is not devoid of challenges. These obstacles include: a) substantial initial investment and ongoing maintenance costs, in addition to the necessity for skilled labor; b) improper operation, which can result in considerable crop damage; c) uneven terrain that diminishes operational efficiency; d) limited effectiveness within crop rows, particularly for densely planted or closely spaced crops; e) the requirement for precise application timing, which is frequently impacted by weather conditions and stages of crop development (Gao and Su, 2024; van der Schans et al., 2006).

Although conventional mechanical weeding techniques work well for large-scale inter-row weed control with little disruption to crops, weeding within rows continues to pose a significant challenge. To overcome this issue, advancements in intelligent mechanical weeding systems have emerged, incorporating smart technologies to improve precision and efficiency. These systems more effectively address intra-row weeds while reducing labor-intensive tasks, providing a promising solution for contemporary agriculture (Quan et al., 2022). The next section delves into the essential elements and innovations that are propelling intelligent mechanical weeding methods forward.

### 3.2 Fundamental elements of intelligent mechanical weeding

**Figure 6:**
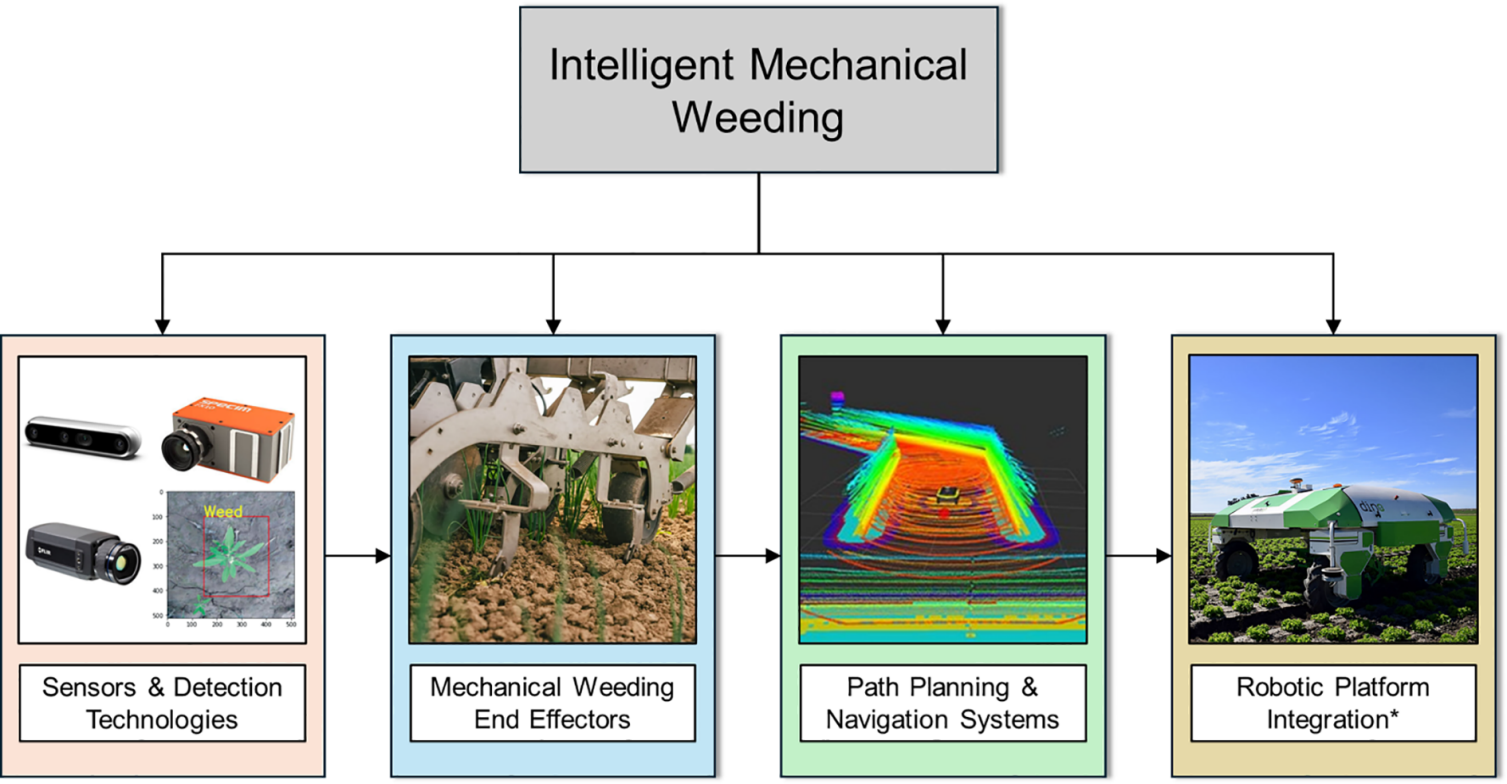
Components of intelligent mechanical weeding. *Dino (Naio Technology).

Intelligent mechanical weeding is an advanced technique that employs technologies such as computer vision, sensors, and precision actuation to detect, identify, and differentiate weeds from crops, enabling targeted weed removal without the use of chemicals (Xiang et al., 2024). Unlike traditional mechanical weeding, which predominantly targets inter-row weeds and relies on generalized tillage or cutting methods, intelligent weeding systems address both inter-row and intra-row weeds with greater precision (Melander et al., 2015). These systems utilize two primary approaches: one involves actively recognizing and removing weeds through automated actuation units, while the other leverages the uniform planting patterns of crops achieved through mechanical sowing to avoid crop interference while targeting weeds (Jiang et al., 2023a; LEMKEN, 2022; Quan et al., 2022). To understand the capabilities of intelligent mechanical weeding, it is essential to explore the key components and technologies, as it has been portrayed in **Figure 6**.

This targeted approach minimizes soil disturbance, reduces labor intensity, and improves efficiency, addressing the limitations of traditional methods, such as poor intra-row weed control and potential crop damage. Intelligent mechanical weeding systems are designed to operate seamlessly in diverse agricultural settings which can adapt to varying crop types and field conditions. Their effectiveness is dependent on the advanced components and technologies that enable precise weed detection, identification, and removal while maintaining high operational efficiency. The following sections will delve into these critical elements in detail.

#### 3.2.1 Sensors and detection technologies

**Figure 7:**
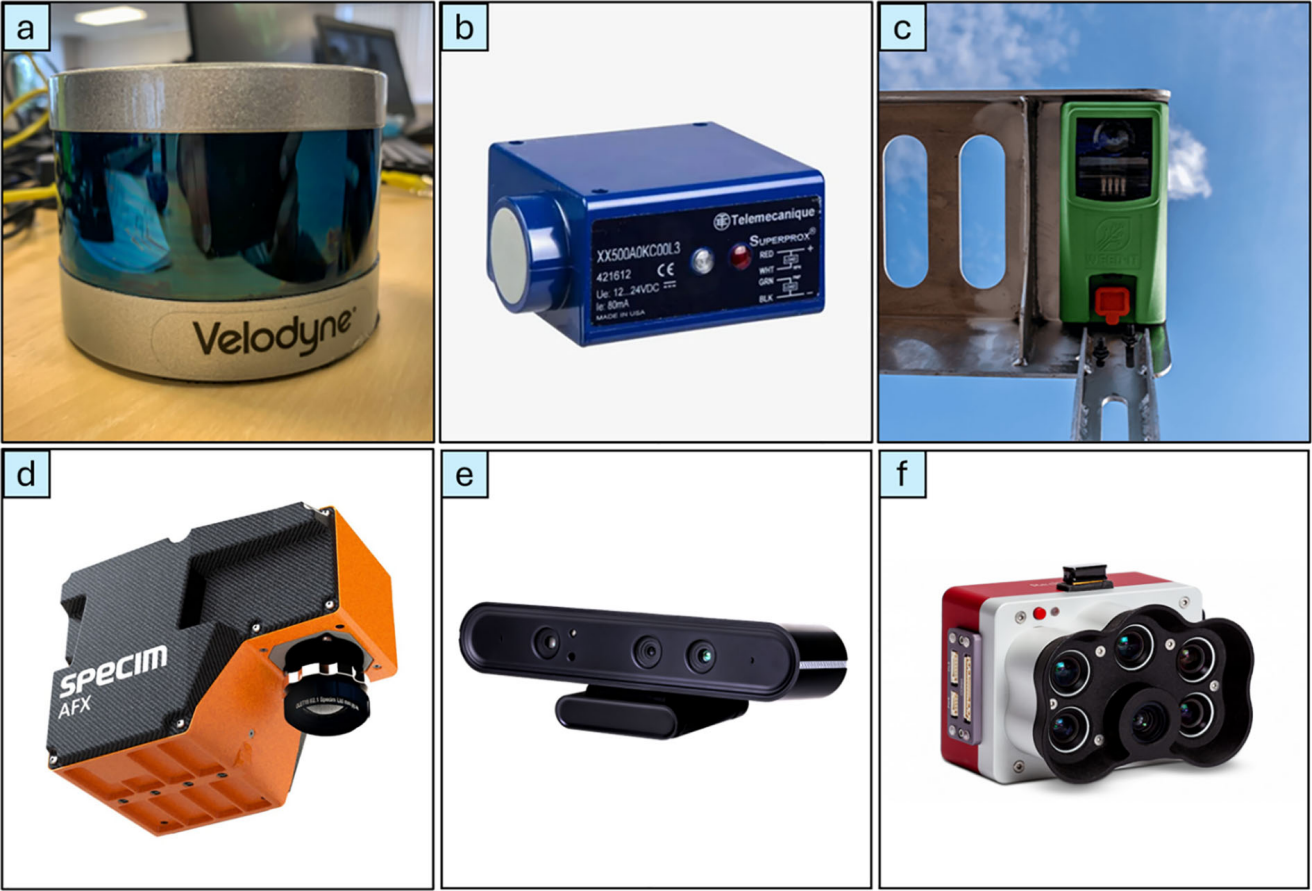
Sensors in use in the intelligent weed management system: **(a)** LiDAR sensor (Collins, 2022), **(b)** optical sensor (Croplands, 2021), **(c)** Ultrasonic sensor (Schneider Electric, 2022), **(d)** Specim AFX17 Hyperspectral Camera (Media, 2022), **(e)** Astra Series RGB depth camera (Vit and Shani, 2018), **(f)** MicaSense RedEdge Panchromatic multispectral camera (MicaSense, 2023).

In intelligent mechanical weeding systems, sensors are pivotal for detecting weeds, enabling precise actuation of the end effector, and navigating environmental obstacles. These sensors facilitate efficient weed management by distinguishing between crops and weeds and ensuring minimal crop damage. The primary types of sensors used in these systems include ultrasonic sensors, optical sensors, laser sensors, imaging sensors, RGB/light sensors, multispectral sensors, hyperspectral sensors, RGB, and depth cameras. Each sensor type contributes uniquely to enhancing the performance of weeding robots, providing crucial data for decision-making and operational accuracy (Upadhyay et al., 2024b). **Figure 7** illustrates commonly utilized sensors in such intelligent weeding systems.

Ultrasonic sensors, which estimate distance by emitting high-frequency sound waves and measuring the time taken for the echo to return, have been effectively used in weed detection, as demonstrated by Andújar et al. (2012), who used vertically mounted ultrasonic sensors to differentiate crops and weeds based on height, achieving reliable detection across samples with varying weed densities. These sensors are light-independent, cost-effective, and adaptable to different environments; however, their performance degrades in wet conditions, and they struggle to differentiate complex plant structures, making them prone to errors in crop-weed distinction and requiring careful calibration (MaxBotix, 2019). Optical sensors detect weeds by analyzing the spectral characteristics of plants through the reflection and interruption of light (Suhail, 2022), as shown in the study by Wang et al. (2001), where classification rates reached 100% for wheat and bare soil and 71.6% for weeds. These sensors are low-cost, fast, and simpler than machine vision systems, making them suitable for real-time weed identification, although their accuracy may vary due to environmental factors and plant spectral variability (Wang et al., 2007). LiDAR sensors operate by emitting laser pulses to create 3D representations of surroundings and are widely used for navigation and weed detection in intelligent weeding robots; for instance, Malavazi et al. (2018) used 2D point cloud-based line extraction to detect crops on the Oz weeding robot. While LiDAR offers high-precision mapping and autonomous navigation capabilities, it requires significant investment, skilled operation, and suffers from limited resolution and environmental sensitivity.

Hyperspectral (HS) and multispectral (MS) sensors, which analyze plant spectral signatures across many wavebands, are powerful tools for weed identification; HS captures narrow, detailed wavebands, while MS provides broader bands and simpler data handling. Graham Ram et al. (2023) utilized hyperspectral imaging with supervised ML models to detect Palmer amaranth weeds, achieving 93.95% accuracy and a 0.95 F1-score, showcasing the strong potential of HS imaging in intelligent weeding. Despite their strengths, HS sensors are complex, expensive, and highly sensitive to lighting, requiring significant data processing, while MS sensors offer a more cost-effective but less detailed alternative.

**Figure 8:**
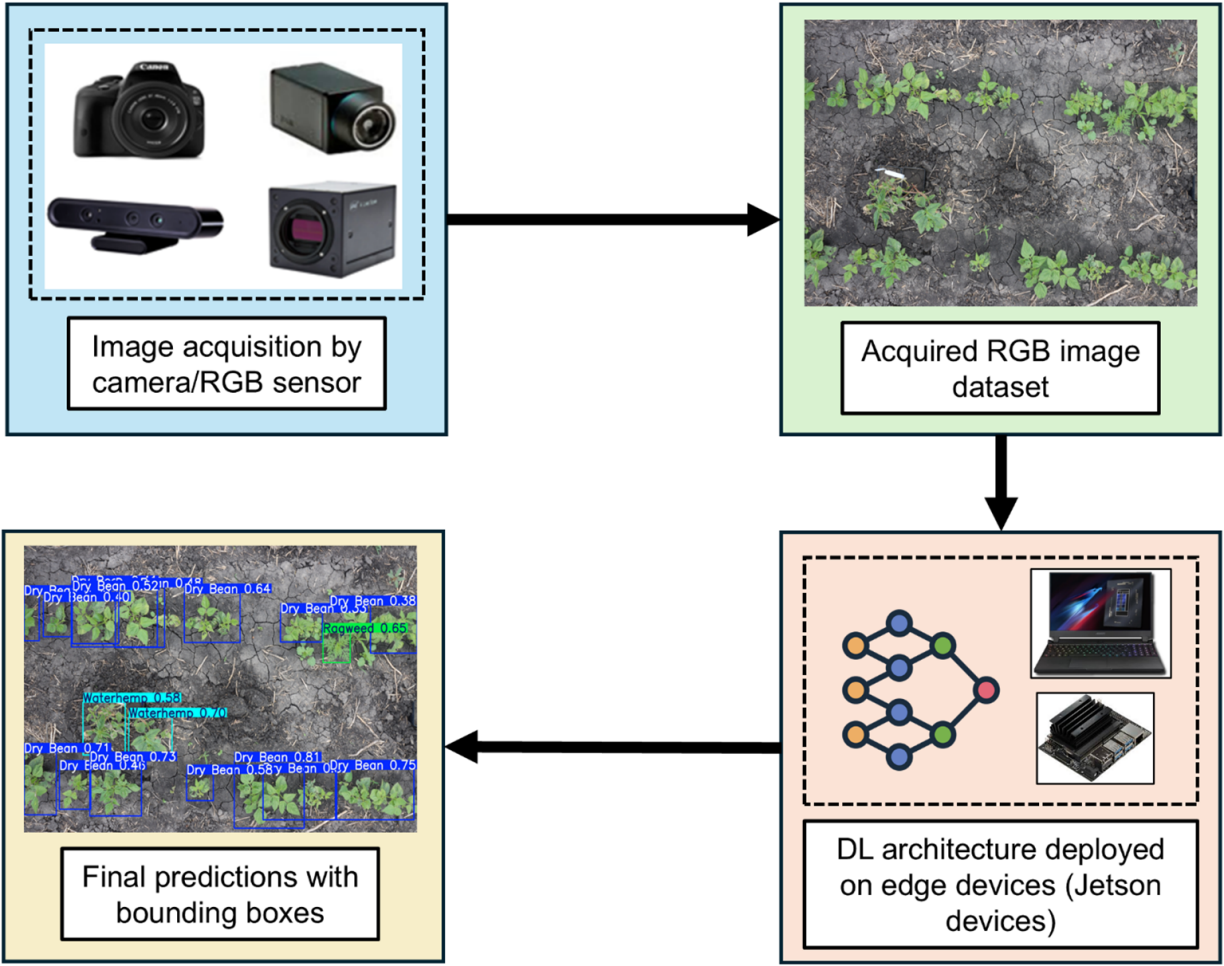
Workflow for weed identification based on RGB images.

RGB/light sensors, including RGB cameras, thermal cameras, and depth cameras, are the most used sensors for crop and weed classification and identification. These sensors capture real-time RGB images from the field as shown in **Figure 8**, enabling targeted weed control and improved crop management.

RGB sensors operate by responding to specific bandwidths within the visible spectrum, primarily the Blue (~450–490 nm), Green (~520–560 nm), and Red (~635–700 nm) bands, to generate color images. Despite their widespread application, RGB sensors are limited to the visible spectrum and are highly sensitive to lighting conditions, which can impact their reliability (Jafarbiglu, 2023). These sensors are often integrated with machine learning (ML) and deep learning (DL) algorithms for weed detection and real-time classification. The general workflow involves acquiring RGB images at specific frame rates with high spatial resolution. These images are then processed through neural networks or detection algorithms, which generate predictions and real-time visualizations. Detection algorithms form the computational core of intelligent weeding systems, transforming raw sensor data into actionable insights. These algorithms typically employ image processing pipelines -including segmentation, feature extraction and classification – to differentiate crops from weeds (Jin et al., 2021; Rai et al., 2023). Classical methods use color indices (ExG. ExR), shape descriptors or texture analysis while modern approaches rely on ML and DL frameworks such as Support Vector Machines (SVM), Random Forests (RF), and Convolutional Neural Networks (CNNs). Advanced architectures like YOLO, ResNet and U-Net enable real-time object detection and semantic segmentation, providing precise spatial coordinates for targeted mechanical actuation (Quan et al., 2022; Rai et al., 2023; Visentin et al., 2023). Integrating these algorithms with sensor data is critical to achieving accurate, fast, and autonomous weed identification in dynamic field environments.

Based on these predictions, the weed removal unit is activated for precise weed eradication (Laftouty et al., 2023). Comparative analysis of multiple deep learning (DL) models including InceptionV3, AlexNet, VGG-16, YOLOv8, and ResNet-50, along with two custom CNN models, revealed that YOLOv8 achieved the highest performance with an accuracy of 100%, while ResNet-50 attained an accuracy of 99%. This study highlights the considerable potential of RGB sensors, particularly when integrated with advanced DL models, for the precise and efficient management of weeds in agricultural settings.

All these sensors play a vital role in weed detection and identification, each operating on distinct principles but sharing the common goal of precise, targeted weed removal. The system’s effectiveness relies on seamlessly integrating these detection technologies with mechanical components that perform the actual weed eradication. The following section explores various mechanical weeding end effectors, the system’s primary interface with the field. A thorough understanding of these end effectors is essential to optimize their compatibility with different detection systems and field conditions.

#### 3.2.2 Mechanical weeding end effectors

End effectors in mechanical weeding systems play a pivotal role as the specialized tools mounted at the tip of the actuation unit, designed to interact with soil and effectively manipulate it to target and destroy weeds. These tools, which can be categorized as either passive or active, are essential for achieving precise and efficient weed control. Their performance and effectiveness are closely linked to the type of actuation unit employed, influencing the way the mechanical tools interact with weeds and soil (Haag, 2021; Xiang et al., 2024).

A comprehensive review of the literature reveals that several mechanisms and implementations have been developed and are in practice. Traditional designs, such as rotary and brush hoes, vertically mounted cultivators, tooth and tine harrows, sweeps, hoe blades, and torsion weeders, operate as passive tools for mechanical weeding (Balas et al., 2022; Stearns, 2021). These tools generally penetrate the soil to depths of 2–4 cm and are primarily used for inter-row weed control. Recent advancements in end effector design emphasize scientific approaches to manipulating soil to uproot, bury, cut, or drill weeds effectively, as noted by Chicouene (2007). These fundamental principles are critical for achieving efficient weed removal in real time. Modern mechanical.

**Figure 9:**
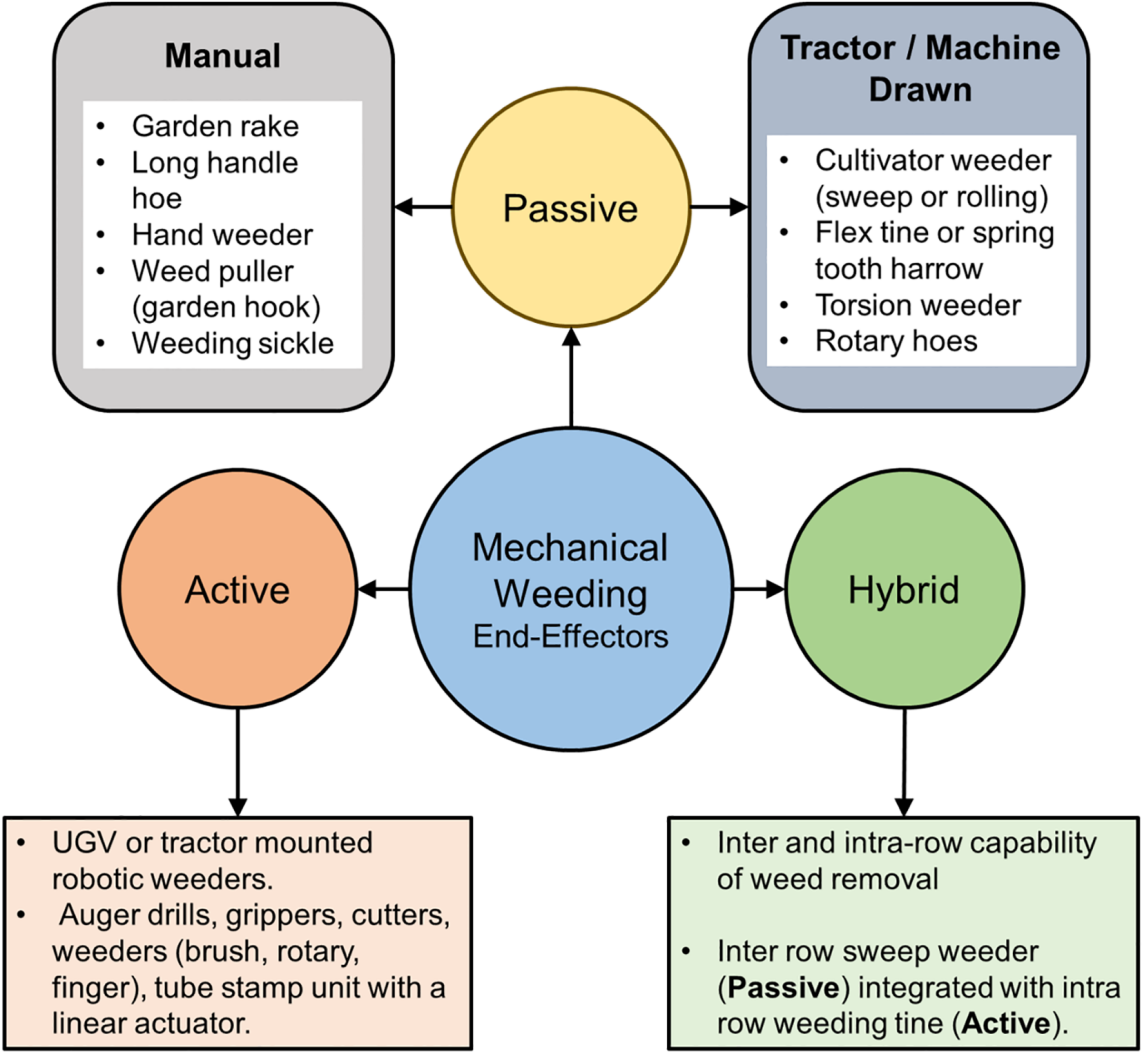
Types of end effectors available and the category (Chandel et al., 2021; Langsenkamp et al., 2014).

Weeding technologies focus on addressing the challenge of intra-row weed control, which is inherently more complex than inter-row weeding. Intra-row weeds grow unpredictably and randomly among crops, requiring end effectors to operate with exceptional precision to avoid crop damage. In contrast, inter-row weeds, which grow between rows of crops, can be managed more effectively using conventional tools with adequate passes (Pradel et al., 2022). To handle the complexities of intra-row weeding, end effectors are being engineered with advanced designs that ensure precise targeting and accurate steering. Calibration and control are critical components in these systems to protect crops while removing weeds efficiently (Reiser et al., 2019). For a generalized understanding, end effectors for mechanical weeding can be broadly classified into three categories: passive, active, and hybrid. This classification encompasses the design principles of the tools, their modes of interaction with soil, and the processes involved in weed destruction. These designs reflect the advancements in the field and underscore the ongoing efforts to develop tools capable of addressing the growing demands of precision agriculture. **Figure 9** illustrates this classification and provides an overview of the diverse end effector designs currently available for mechanical weeding.

**Figure 10:**
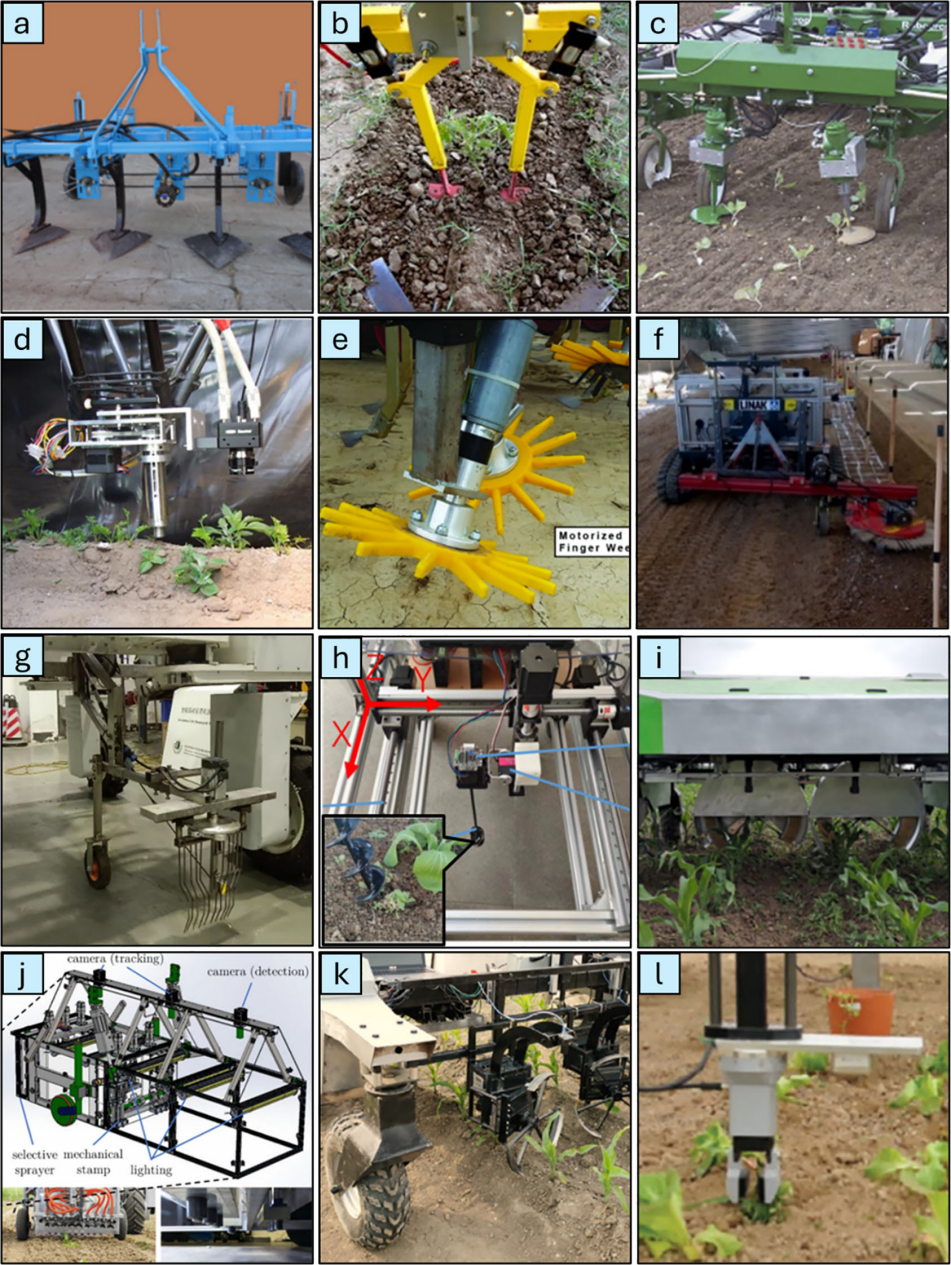
Different end effectors developed for mechanical weeding: **(a)** IIIR weeder (Chandel et al., 2021), **(b)** weeding knives (Kennedy et al., 2020), **(c)** rotary cultivator (Tillett et al., 2008), **(d)** tube stamp weeder (Langsenkamp et al., 2014), **(e)** Finger weeder (Machleb et al., 2021), **(f)** brush weeder (Reiser et al., 2019), **(g)** reciprocating elastic comb weeder (Ye et al., 2023), **(h)** drill end effector (Wang et al., 2024), **(i)** weeding brush (Jiang et al., 2023b), **(j)** mechanical stamp (Wu et al., 2020), **(k)** disc weeding knife (Quan et al., 2022), **(l)** robotic gripper weeder (Visentin et al., 2023).

**Figure 10** demonstrates various types of end effectors developed for mechanical weed removal, designed to address both inter-row and intra-row crop scenarios. These designs are tailored based on the size and growth stage of the weeds being targeted. Weeds in their early growth stages can be selectively eradicated using robotic end effector tools engineered for precision removal. Conversely, intra-row weeds that are distributed extensively across the soil surface often require continuous operation tools, such as rotary cultivators or weeding knives. These tools are designed to manipulate the entire soil area between crops consistently, ensuring comprehensive weed removal. The selection of an appropriate end effector is influenced by several critical factors, including the size and type of weeds, the prevailing soil conditions, and the required frequency of operation. This specificity ensures optimal performance of the weeding system and minimizes the risk of crop damage while achieving effective weed control (Asaf et al., 2024; Zawada et al., 2023).

For effective and intelligent mechanical weeding, integrating sensing and detection technologies with actuated removal units is crucial. These systems operate together, where sensing and detection technologies identify and distinguish weeds from crops, while the actuated removal units perform precise mechanical actions to eliminate the targeted weeds. The integration required real-time synchronization between detection outputs and the end effector responses to ensure precise targeting and minimal crop disturbance. Building on the foundation of sensing and detection systems and the various types of end effectors discussed earlier, the next step in advancing precision weed management focuses on integrating these tools with robotic platforms. This integration not only enables autonomous operation but also ensures adaptability and efficient handling in different field conditions. The details of this transformative approach are examined in the following section.

#### 3.2.3 Vision guided actuation and system integration

**Figure 11:**
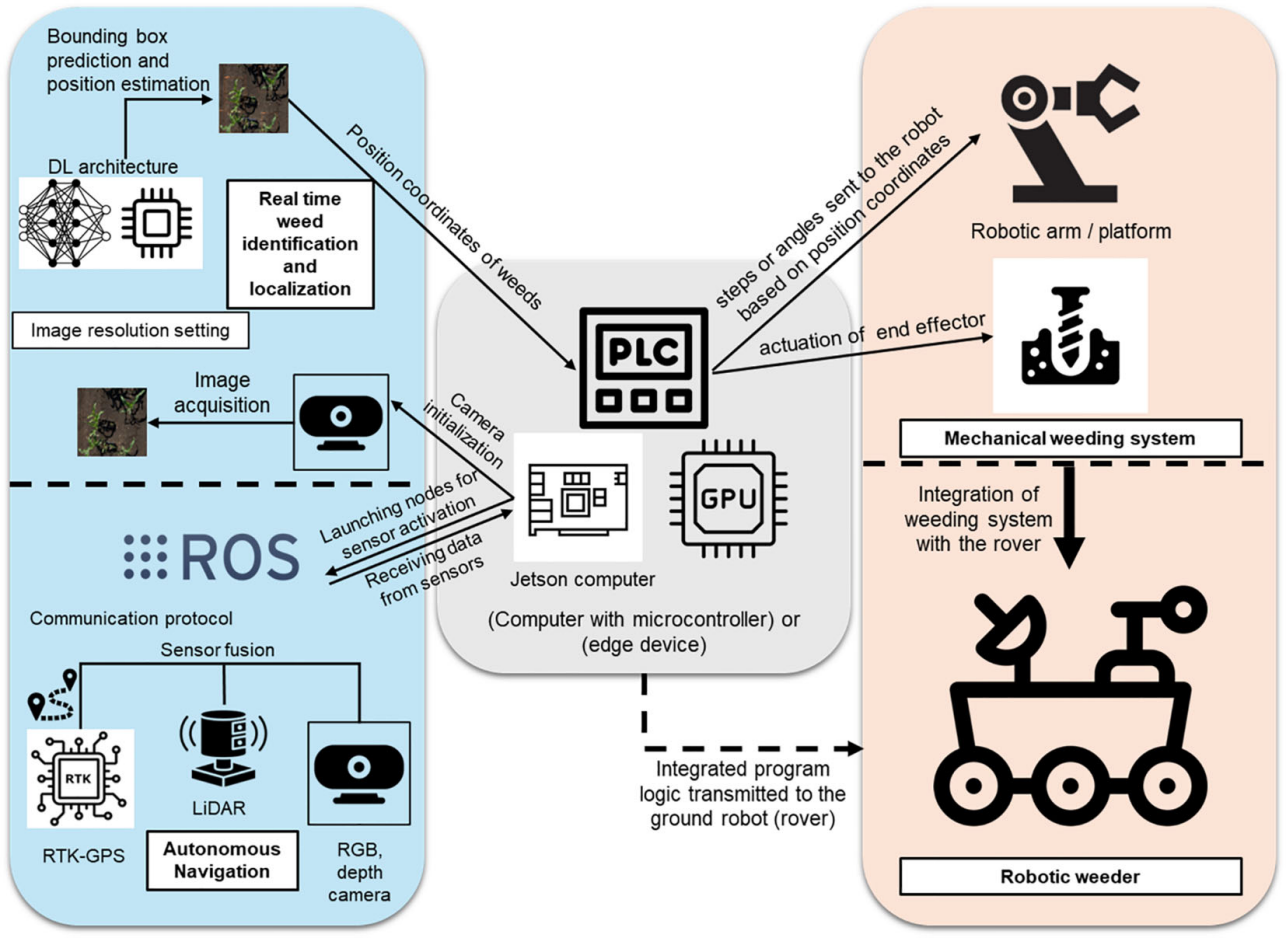
Schematic representation demonstrating the integration with the robotic platform for intelligent mechanical weeding systems.

In conventional agricultural practices, mechanical weeding has traditionally been carried out either manually or by integrating conventional mechanized equipment with tractors. This method, while effective, heavily relies on the precise calibration of the equipment and the professional expertise of the operator (Hussain et al., 2018; Machleb et al., 2020). Consequently, their implementation is often labor-intensive and associated with high operational costs. To address these limitations, the adoption of autonomous methods for mechanical weeding has emerged as a promising and reliable alternative. These methods not only facilitate intelligent weed eradication but also significantly enhance operational efficiency (Liu et al., 2023). Autonomous weeding machines, often referred to as weeding robots, operate based on predefined rules and program logic. The primary objective of these systems is to accurately identify, classify, and localize weeds. Once detected, the integrated actuation unit removes the weeds autonomously, guided by the programmed algorithms. These systems represent a convergence of artificial intelligence (AI), robotics, and agricultural technology, forming a robust solution for precision weeding (Bernier, 2024). To establish a robotic platform dedicated to mechanical weeding, its components must be systematically organized to ensure efficient operation. **Figure 11** provides a detailed illustration of the operational workflow of a ground robotic platform designed for mechanical weeding. For example, Visentin et al. (2023) developed a robotic platform capable of addressing both intra-row and inter-row weed removal, featuring operational and control architecture comprising key components. First, sensing and weed detection is achieved using an RGB sensor or camera that captures real-time field images, which are then preprocessed for weed detection. ML or DL-based algorithms analyze these images to determine the precise positions of weeds. Second, a programmable logic controller (PLC), which may include a standalone microcontroller, computer, or edge computing devices such as Jetson or Raspberry Pi, processes the detection data. These systems execute real-time computer vision algorithms and generate commands based on detection logic. Finally, the processed commands are transmitted to the mechanical actuation unit, responsible for the precise removal of weeds, operating in real-time to ensure accuracy and efficiency. This diagram encapsulates the systematic flow of data from sensing to actuation, highlighting the integration of advanced technologies for autonomous weed control.

This is a basic workflow that is followed by robotic weeders for real-time autonomous mechanical weeding. There are several weeding robots and mechanical weeding systems which have been developed so far, some of which are research-based, developed by some organizations, while others are commercially available, industry-grade. For instance, a work conducted by Chang et al. (2021) used a deep convolutional neural network for weed identification and localization, and a pyramid-shaped shovel-like end effector for effectively uprooting the weeds from the soil. The system has been tested by being operated at a speed of 20 cm/s and has proved to be effective with a 92.6% success rate in weed removal. In a study by Shanmugam and Asokan (2015), a machine vision-based mechanical weeding system was developed for turmeric fields, utilizing a robotic arm controlled via a MATLAB platform. The system achieved a weed-plant separation accuracy of 94%, with the ExG-ExR method further enhancing separation accuracy to 98%.

**Table 2** provides a comprehensive analysis of various advanced mechanical weed management systems that have been developed over the years.

**Table 2:** Descriptive analysis of the intelligent mechanical weed management systems.

Robot/weeding platform	Sensors/software	Weed identification method	Mechanical weeding mechanism	Crop	Performance	Reference
Intra-row weeding system	Industrial camera	SPH-YOLOv5x model for crop detection	Real-time weed knife control system	lettuce	Weed identification model accuracy of 95%, mAP value of 96% and weed removal accuracy of 80.25% at 2.38 km/h	Jiang et al. (2023a)
Intra-row weeder	Ultrasonic sensor	Ultrasonic sensor-based plant detection	Hydraulic actuated pinch roller weeding mechanism	Cabbage	Plant detection accuracy has R^2^ value of 0.94, and the weed-specific accuracy of 33.9% for Southern crabgrass	Saber et al. (2015)
Odd Bot Maverick	High-resolution RGB camera and 3D depth camera	AI-driven vision-based weed detection system	Two delta arms with grippers for pressing and pulling out weeds	Carrots, Onions, & Chicory	Precise weed removal with an accuracy of 2 mm and operational speed of 2 weeds/sec at 0.6 km/h	Odd.Bot (2024)
Smart weeding machine	Digital camera	YOLOv3-based weeds detection and localization	DC motor-driven claw rake weeding tool	N/A	Weed detection accuracy of 95.6% at 5 frames per second. At 15 cm/s weeding success rate of 92.6%	Chang et al. (2021)
BoniRob	Camera for visual servoing	Human image processing and position transfer via mobile network	Linear actuated tube stamp	Maize	Weed control rate of 93.86% was attained	Langsenkamp et al. (2014)
Weed Spider Robotic Weeder	LiDAR, GNSS, camera	LiDAR-based weed mapping	Mechanical weeding arm with blades and automatic depth control	Tobacco, sweet potatoes, soybean	95% reduction in labor costs and works up to 3.5 acres per hour	GreenTech Robotics (2023)
Intelligent intra-row weeding robot	Industrial grade camera module (RER-USBFHD01M-LS36, Shenzhen, China)	YOLOv5 network selected as the vision system	Weeding brush fitted with wire brush and brush rollers with roller support	Maize, Chinese cabbage	Weed removal rate in maize and Chinese cabbage 90% and 94.5% respectively and crop damage of 1.9 and 0.8%	Jiang et al. (2023b)
AgBotII	RGB and NIR camera (IDS UI1240SE 1.3MP global shutter camera)	Vision based online detection and classification based on color spaces	Robotic Blade hoe	Cotton, wild oats & sow thistle	Weed classification accuracy was 96%. Highest performance in cotton (97.8%)	Bawden et al. (2017)
Rover	Intel RealSense D435i, RGB-D camera	CNN based on ResNet18, part of PlantNet	3D printed claw gripper on a gantry robot	Lettuce	Crop and weed detection accuracy above 97% and effective weed removal ~85% with crop damage less than 5%	Visentin et al. (2023)
Universal mobile robot	Industrial USB digital camera (6-DZM-12, PHZL Co., Ltd., Shenzhen City, China)	YOLOv3 network for real-time weed detection	Disc weeding knives (blade, wedge, and plough) with arbours	Maize	Weed removal rate of 85.91% and crop injury of 1.17% and YOLOv3 detection accuracies for maize and weeds were 98.5% and 90.9% respectively	Quan et al. (2022)
Phoenix	2D laser scanner, Electromechanical sensor and sonar sensor	Trunk position detection based on the sonar	Electric rotary weeder	Vineyard	Average tilled area was 65% for feeler and 82% for sonar	Resier et al. (2019)
Tertill	Capacitive sensors	Capacitive sensors detect tall plants and to avoid obstacles	Four camber wheels (grousers), weed whacker	Pearl millet	Efficiency ranged from 54-75%	Sanchez and Gallandt (2021)
Weeding robot	Laser ranging sensors (BL-200NMZ)	Laser ranging sensor-based plant position estimation	Reciprocating elastic comb	Soybean	Weeding rate of 98.2% and crop injury of 1.69% at optimal speed of 0.31 m/s at 29.06 mm depth	Ye et al. (2023)
Modified BoniRob	Global shutter camera (JAI AD‐130 GE) and 8 mm lens (Fujinon TF15‐DA‐8), narrow‐beam sonars (SRF235 Ultrasonic Range Finder)	Naïve Bayes filtering, intra and inter camera visual tracking	18 stamping tools composed of pneumatic cylinder	Sugar beet	Operated at 0.05 m/s on flat and rough terrain efficiency was 99.11% and 99.17% respectively	Wu et al. (2020)
Robovator	Camera	Image processing-based binary segmentation	Torsion weeder with square tines	White cabbage	Intelligent weeding can remove weeds closer to crops without subsequent manual weeding	Melander et al. (2015)
Mechanical weeder	Red-infrared camera,	Machine vision system with the software IMPASS	Motorized finger weeder	Sugar beet	Intra row weed control efficacy ranged 87 to 91% in 2017 and 91 to 94% in 2018	Machleb et al. (2021)
Tractor- intra row weeding platform	color camera (Do3think CM036) and lens (AZURE-0420mm)	Image processing algorithm consisting of thresholding, refinement and filtering	C-type vertical axis weeding blade	Cauliflower, lettuce and maize	Identification rates of crops were above 95% and operating speed of 2 km/hr improves efficiency 34.4 times of a manual labor	Nan et al. (2015)
Camera guided hoeing system	Two contact sensors, 3 m wide cameraguided hoeing system (K.U.L.T.-Kress Umweltschonende Landtechnik, Kürnbach, Germany)	RGB camera for crop row scan and analysis and hoe alignment adjustment with contact sensors	Hoeing implement (sweep) with contact disc	Maize	demonstrates effective slope force compensation on various gradients, enabling precise hoeing on sloping terrains	Spaeth et al. (2024)
Tractor with hoe Chopstar	2D RGB camera	Colour and height-based camera setting adjustment	Row hoeing with chopstar, post emergent hoeing goosefeet sweeps	Sugar beet	Interrow weed control efficiency was between 94-98% for the goose feet sweeps	Parasca et al. (2024)
Tractor- camera steered mechanical weeder	OEM Claas 3-D stereo camera	3D camera and artificial lightning-based row detection for weeding hoe automatic steering, crop identification based on size	Duck foot blades for inter row with four different intra row weeders- flexible finger weeder, torsion weeder, rotary harrow or ridging blades	Maize, Soybean, Sugar beet	Camera steered hoeing had an efficiency of 78% and yield increase in white sugar, maize and soybean by 39, 43 and 58% respectively.	Kunz et al. (2018)
Tractor- rotating vertical tines	RGB-D sensor (Kinetic version 2), photonic sensor for depth estimation	Computer vision and image processing including feature extraction	Spinning tines mounted on pivoting arms with servo motors	Broccoli & lettuce	Segmentation accuracy was in the range of 87.2 to 96.6% for broccoli and 74.12% to 92.4% for lettuce.	Gai et al. (2020)
Robotic weeder	Digital color camera (Model piA240012gc) with fixed focal length lens (M0814-MP2–8 mm)	Geometric appearance-based crop detection	Robotic weeding knife	tomato	Tomato stems detected with 99.19% accuracy	Raja et al. (2020)
Self-propelled inter row weeder	Camera	Visual recognition system for path planning	Weeding wheels with rake teeth	Paddy	At a forward speed of 0.64 m/s, weed rate prediction accuracy was 88.43%	Tang et al. (2021)
Tractor with harrow	5 MP RGB camera and 25 mm lens, RTK-GNSS receiver	AI algorithm and DL-based weed/crop cover detection	Weeding harrow tines	Barley	R^2^ prediction value of 95.9% for weed cover and 98.6% for crop cover in pre-harrow images and 88.4 and 97.7% for post harrow images	Berge et al. (2024)
Classification based robotic weed control	industrial camera (HF868-2)	LettWd-YOLOv8l model for object detection	Weeding knives	Lettuce	Achieved 99.73% precision and 99.5% F1-score on indoor dataset under varied lighting, intra-row weeding rate of 83.7% at 3.28 km/h	Zhao et al. (2024)
Precision mechanical weeder	Camera	Deep learning-based weed detection and signal-based weeder actuation	Spiral bar type weeding head	Corn	95% weed removal rate and 3% crop at a movement speed of 80 mm/s	Hu et al. (2024)`
Pneumatic precision seeder Optima V	Camera (KULT iVision PV)	Vision based Row detection	Cutting discs, no till sweeps,	Maize	Bidirectional hoeing increased efficiency from 80% to 95%	Naruhn et al. (2023)
Intra-row weeder	Ultrasonic sensor (28015 PING), proximity sensors (18–14 DP2)	Crop weed sensing with position sensor	Vertical axis rotary shaft with weeding blade	Green chili & tomato	Effective weed control efficiency above 65% and crop damage less than 25%	Kumar et al. (2020)
FarmDroid FD20	RTK-GPS	Early-stage weeding based on high precision GPS technology	6 hoeing tools	Sugar beet	Increase in sugar beet yields from 40-60%	Rossmadl et al. (2023)
Mechatronic Intra-row weeding system	CMUcam5 Pixy camera	Vision based crop detection and localization	Crescent shaped blade	Corn	Demonstrates effective crop recognition and weeding under controlled conditions	Berkmortel et al. (2021)
BonnBot-I	Camera (Intel RealSense D455), Inertial Navigation System	Instance segmentation based on Mask-RCNN	Mechanical hoeing	Corn	The Normalized Absolute Error reduced from 8.3% to 3.5% for the weeding platform	Ahmadi et al. (2022)
Weed hoeing	RGB camera	Image segmentation. Background separation by Kalman filter	No-till sweeps	Winter wheat	Camera guided weeding efficiency 72-96% for inter-row and 21-91% for intra-row	Gerhards et al. (2020)
Agrobot	HP webcam (W100HP) – 1280 ×720 resolution	Image processing and DL-based weed identification; MobileNetV2 and SSD FPN-Lite	15 cm wide shaped blades (inter row); 15 cm flat sharp blades (intra row)	N/A	Weed detection model accuracy of 99%	Jog and Agashe (2024)

Based on these research studies, it has been observed that most of the works have focused on accurately detecting the weeds in real-time field conditions and using custom end effectors for weed removal. The idea of integrating these detection models with the Program Logic Controller or edge devices for accurate robot actuation is a challenging task. The main concern lies in getting the exact position of the weed from the detection models and synchronizing them with the inverse kinematics or the actuation unit of the robotic system for real-time weeding.

**Figure 12:**
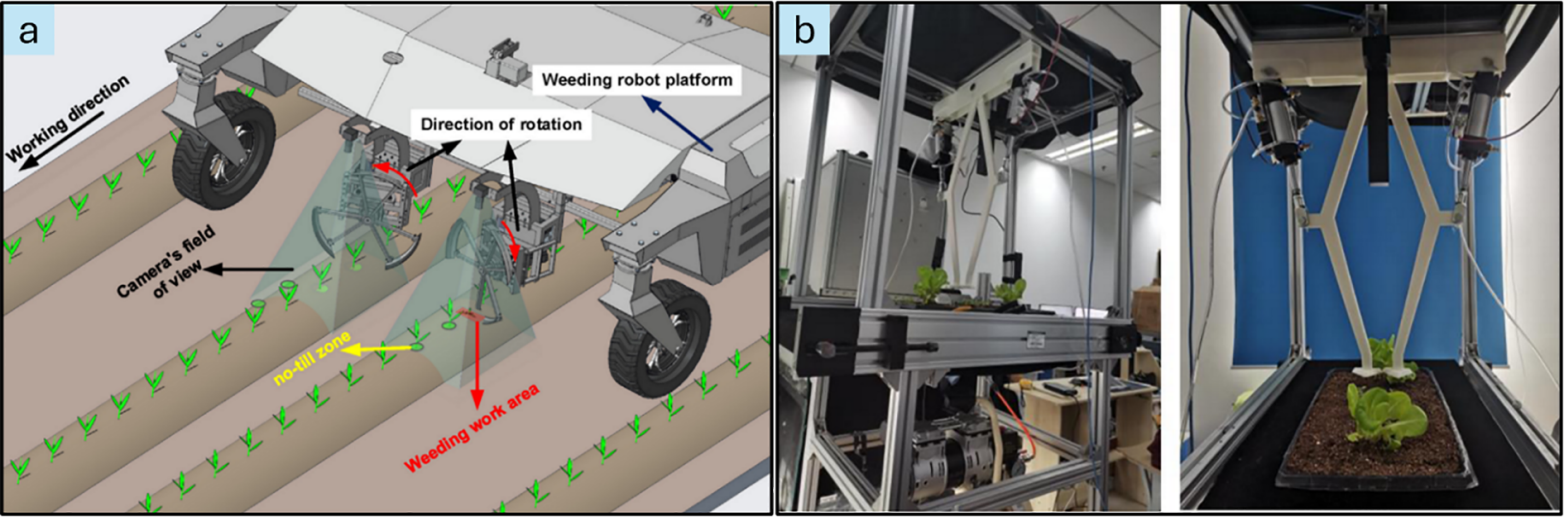
Vision-based weed removal without object localization **(a)** Intra-row weeding platform using camera and rotating disc weeding knives (Quan et al., 2022), **(b)** experimental setup on artificial soil bin with weeding knives control system. (Jiang et al., 2023a).

**Figure 13:**
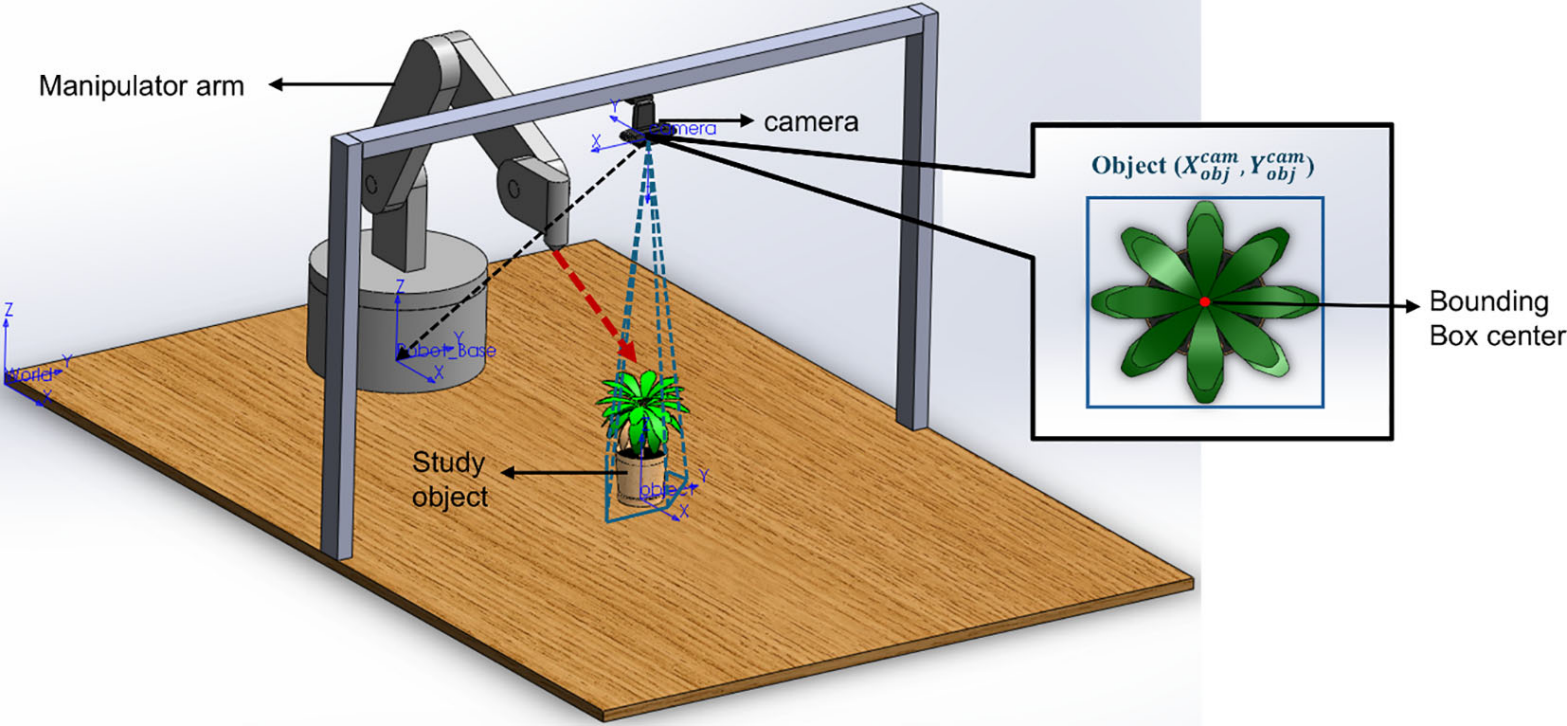
Object localization for coordinate estimation and robot actuation.

In the context of weeding, there are two cases based on which the robotic system removes the weeds. Depending on the degrees of freedom of the automated unit, the conceptualization of the actuation logic is determined. Firstly, the robotic weeder with an actuation unit with a single degree of freedom has a simple operational logic for removing the intra-row weeds based on detecting the crops under consideration at the time of operation. **Figure 12** shows examples of the operation of the automated vision-based weeding unit that does not require localization to eradicate weeds; rather, it detects the presence of crops and tills the intermediate soil area. A camera mounted near the actuation unit detects the presence of crops, and in turn, the actuation unit operates to remove the weeds in no-crop regions and vice versa. This type of actuation is effective if the position of the weed is uniform; otherwise, depending on the design and the structure of the contact tool, there are chances of large amounts of unnecessary soil manipulation. Secondly, another approach to automated and robotic weeding involves the use of a robotic arm for the purpose. The concept of weed detection and localization for the actuation of robotic arms is the same for all the types of robots that are available. **Figure 13** shows an experimental setup with a robotic manipulator arm and a camera mounted over the target object for real-time coordinate estimation and actuation.

The main complexity in using a robotic arm for weeding lies in estimating the position of the object in real time with respect to the robot base. In practice, detections arrive in the camera frame. For inverse kinematics (IK), however, the target must be expressed in the robot base frame. Suppose the detected bounding-box center is $\left(X_{obj}^{cam},Y_{obj}^{cam}\right)$(and depth $Z_{obj}^{cam}$ when available), the homogenous point in the camera frame is represented as

(1) Pobjcam=[XobjcamYobjcamZobjcam1]

To express the object in the base frame, pre-multiply by the homogeneous transform of the camera with respect to the base (Cao et al., 2019).

(2) Pobjbase= Tcambase .  Pobjcam

Here Pobjbase refers to the position of the object with respect to the base of the robot and Tcambase represents the homogeneous transformation matrix (Briot et al., 2015) for frame transformation from the camera frame to the base frame. The transform has the standard block form

(3) Tcambase=[Rt01×31],  R∈SO(3),  t∈ ℝ3  

Equations 1–3 define the full transformation pipeline that maps detections from the camera frame into the robot base frame, enabling IK-based actuation. Where R and t are the camera’s orientation and position expressed in the base frame (ontained from hand-eye calibration or kinematic calibration). After applying (2), the arm can attempt IK to reach Pobjbase if the point lies with the reachable workspace. This pipeline exemplifies the integration of perception and manipulation for precise, sustainable, and efficient weed management.

As illustrated in **Figure 10**, the overall performance of an autonomous weeding system is substantially affected by its locomotion capabilities. Consequently, the following section explores the path planning and navigation systems that are essential for effective robotic operation in agricultural fields.

#### 3.2.4 Path planning and navigation systems

**Figure 14:**
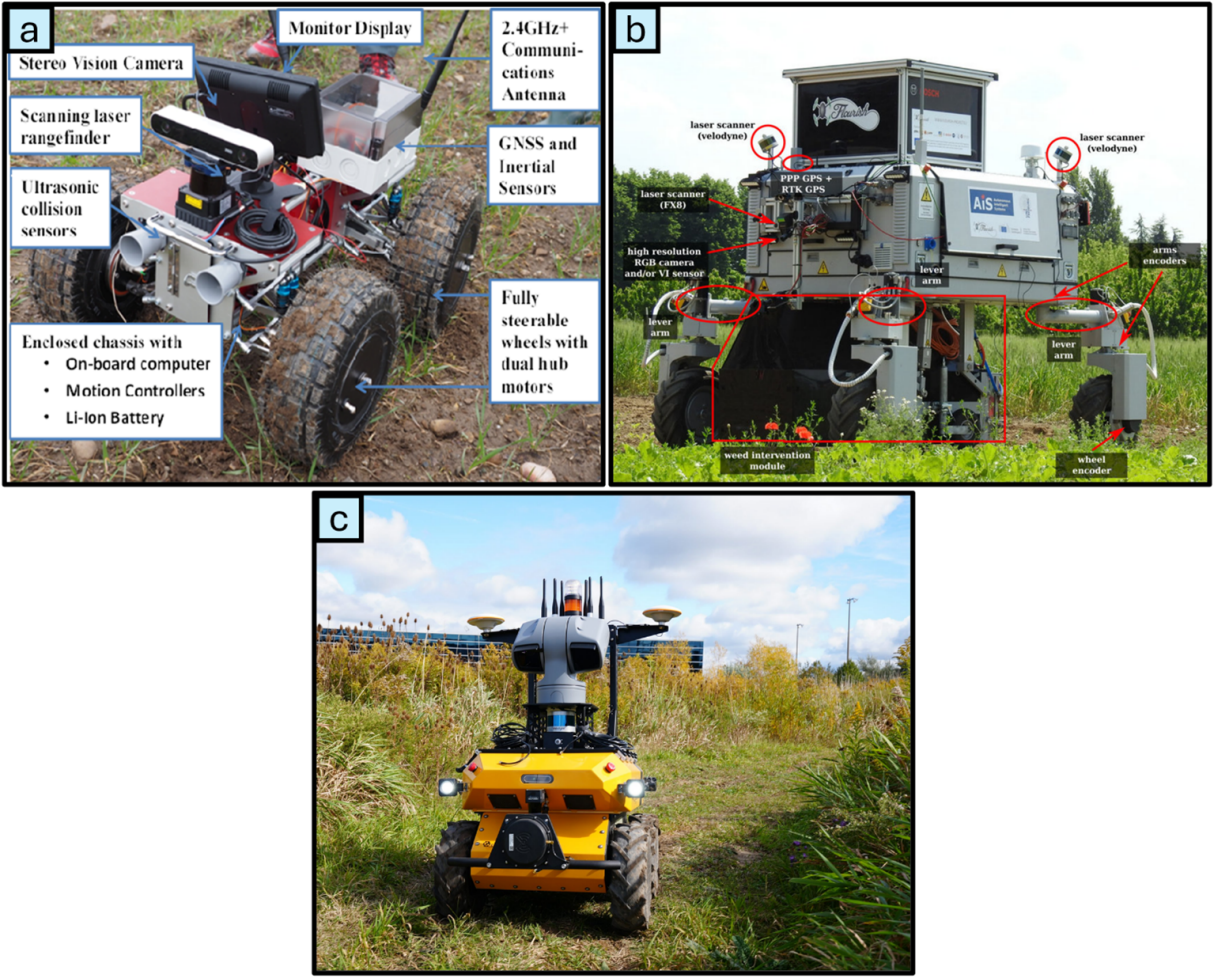
Multiple sensor-based robotic platforms: **(a)** Rover platform (Post et al., 2017), **(b)** BoniRob farming UGV (Pretto et al., 2021), **(c)** Husky Observer (Clearpath Robotics, 2023).

For autonomous and precise mechanical weeding in real-time field conditions, robots must possess precise positioning capabilities and the ability to autonomously navigate fields intelligently. Robot navigation relies on the ability to continuously determine its real-time position and orientation, enabling path planning and collision avoidance (Shalal et al., 2013). Various navigation systems have been developed, integrating sensor-based technologies, computational methods, and control strategies as shown in **Figure 14**. Accurate navigation is fundamental to operational efficiency, as it requires continuous positional tracking and dynamic adjustments in movement to effectively address the challenges inherent in unstructured agricultural environments.

Agricultural navigation systems face several challenges, including unstructured terrains, environmental noise, and hardware limitations such as wheel slippage and end effector failures (Yao et al., 2024). GPS provides precise ground vehicle location data through satellite-based positioning, as demonstrated in a study by Mwitta and Rains (2024), who integrated GPS with visual navigation for cotton field operations. Their system achieved a lateral deviation of 4.8 cm from the desired path, showcasing the potential of GPS-augmented solutions. However, GPS sensors face challenges in closed environments like indoor conditions, where satellite signal obstruction and weather-induced noise affect performance. The inclusion of vision sensors has been shown to enhance system accuracy under such conditions (Binbin et al., 2021). LiDAR (Light Detection and Ranging) is another key sensor used in agricultural robot navigation, leveraging laser pulses to measure distances and mapping the environment in real-time. LiDAR sensors facilitate obstacle detection and avoidance, as demonstrated by Aarab (2023). For example, Abanay et al. (2022) developed a navigation system for the AgriEco Robot using a 2D LiDAR sensor integrated with ROS, achieving a lateral error within a few centimeters and an RMS error of 2.99 cm at a navigation speed of 0.44 m/s. Similarly, Malavazi et al. (2018) utilized LiDAR-based line extraction from two-dimensional point clouds, utilizing the PEARL method and successfully applying the algorithm to the Oz weeding robot.

Relying on a single sensor for navigation can result in reduced accuracy and increased path deviations. Sensor fusion, which combines data from multiple sensors, such as LiDAR, cameras, ultrasonic sensors, and RADAR, addresses these limitations and improves navigation performance (Rigoulet, 2021). For instance, Velasquez et al. (2022) developed an autonomous navigation system integrating LiDAR for crop row detection and IMU for enhanced performance.

The system underwent testing over 50.88 km in various field conditions, achieving average distances between interventions of 386.9 m in gap-free fields, 56.1 m in production fields, and 47.5 m in fields with 1 m gaps, demonstrating robust performance in diverse agricultural environments. Upadhyay et al. (2024b), have provided a comprehensive review of this navigation approach offering valuable insights into the design and implementation of advanced agricultural robotics.

## 4 Technical challenges and future directions

The primary objective of a precise mechanical weeding system is to effectively eliminate and manage weeds while minimizing environmental impact, reducing crop injury, and ultimately enhancing net yield. These goals form the foundation for designing and developing an efficient weed management system. Moreover, economic feasibility and platform versatility are crucial factors that influence the adoption of these systems by farmers and stakeholders. These elements play a vital role in motivating users to invest in advanced weed management technologies. Based on an extensive literature review and analysis of documented research examples, it is clear that significant progress is still needed to optimize the performance of such systems to fully realize their potential.

Currently, the most commercially available mechanical weeding systems are tractor-mounted implements designed for traditional weed management methods. However, the adoption of intelligent and autonomous mechanical weeding technologies remains in its early stages. Farmers and end-users are gradually becoming familiar with these advanced systems. Despite significant progress in autonomous agricultural systems, including deep learning and machine vision-based weed detection, several limitations and challenges persist.

Technical challenges in autonomous mechanical weeding systems include precise identification and localization of weeds in real-time scenarios. Although cutting-edge deep learning models, such as the latest variants of the YOLO (You Only Look Once) framework, have shown excellent performance in weed detection and classification, several challenges remain. These challenges encompass issues related to dataset quality and requirements, variations in lighting conditions, morphological similarities between crops and weeds, overlapping images in datasets, and the dynamic nature of environmental conditions. Additionally, the different growth stages of weeds further complicate detection tasks. Beyond these data-related challenges, computational costs, hardware limitations, and potential misidentifications or incorrect detections present significant hurdles. For instance, inadequate datasets or insufficient model training can lead to performance degradation in real-world scenarios, particularly under varied environmental conditions (Rai and Sun, 2024; Saiwa, 2024; Wang et al., 2019).

To address these limitations, deep learning models should be trained on larger and more diverse datasets enhanced through augmentation techniques, such as brightness adjustments, noise addition, and rotation, to simulate real-world variations. Furthermore, the model architecture implemented on edge devices must prioritize lightweight designs to reduce computational complexity while preserving accuracy. Striking a balance between model performance and processing speed is essential in selecting the optimal model for real-time weed detection tasks. By ensuring this trade-off, the models can achieve higher accuracy and efficiency in field operations, ultimately leading to more effective and reliable weed management solutions.

Another major challenge in autonomous mechanical weeding systems is the potential damage to crops caused by the physical interaction of mechanical tools with the soil during weeding operations. This issue is critically important, serving as a key selection criterion for any mechanical weeding system. While the primary aim of these systems is to eliminate weeds, unintended harm to crops can reduce yield, thereby undermining the system’s effectiveness. Although inter-row weeders generally operate between crop rows and pose minimal risk to crops, the concern increases with intra-row weeders, which operate in closer proximity to the soil and crops.

The weeding end effector analyzed in this review exhibit a diverse array of operational methodologies, each characterized by distinct levels of efficiency and an associated percentage of crop injury. At present, crop injury and inadvertent interactions, arising from misidentifications, variations in weed morphology, and diverse soil types, continue to pose challenges that have not yet been thoroughly addressed. The design of end effector capable of precise targeting with minimal impact on crops represents an area of ongoing scholarly inquiry. Approaches designed to tackle these challenges encompass the advancement of high-resolution weed mapping techniques, facilitating the creation of an accurate real-time distribution of both weeds and crops, thereby clearly delineating the operational territory for the end effector. Furthermore, the designs of end-effectors ought to be adaptable to various weed growth stages and soil conditions.

To tackle the challenges related to autonomous mechanical weeding systems, the manufacturing and research sectors must follow a comprehensive set of guidelines and standards. These guidelines should act as a benchmark for creating effective and broadly acceptable systems capable of achieving precise weed eradication and sustainable management. The following points outline the essential requirements for the proper functioning and operation of weed management systems:

a. A compact, efficient, and lightweight detection model should be chosen for the task of weed identification and localization. These models must undergo rigorous testing in both controlled and field environments before deploying on-edge devices to ensure reliable performance in real-world conditions.
b. The weeding tools must be specific to crops or adjustable, allowing them to accommodate environmental variability, including changes in weed morphology, soil types, and topographical conditions. This adaptability ensures the system’s effectiveness across various agricultural settings and crop types.
c. The developed system must have a robust and adjustable design, regardless of geographical location or crop type. It should allow for flexible operation, functioning either as an independent unit or as a dependent unit mounted on a tractor. For example, mechanical weeding systems should be capable of operating autonomously as robotic platforms or integrated with tractors for simultaneous weeding and other intercultural operations (GreenTech Robotics, 2023).
d. The components—such as sensors and computing devices—that make up the robotic mechanical weeding system should be selected and integrated to balance high performance with economic feasibility. Additionally, the system’s control logic must include a self-reset feature to recalibrate the platform to its default settings in case of operational disruptions during fieldwork.
e. The system should incorporate an intuitive and user-friendly interface, enabling farmers and operators to easily understand the system’s operations. The interface should also assist users in diagnosing and resolving issues that may arise during field operations in real time.
f. To improve the navigation accuracy of the robotic platform, sensor fusion should be utilized. This means integrating multiple sensors, including LiDAR, ultrasonic sensors, RTK-GPS, and RGB cameras, to offset the limitations of individual sensors. The combination of these technologies guarantees precise localization and reliable performance across varying environmental conditions.

While mechanical weeding serves as a reliable option for effective weed management, an integrated method that combines mechanical systems with advanced techniques such as laser-based weed removal and “see-and-spray” technologies can enhance performance even further. This hybrid strategy provides intelligent application capabilities while emphasizing environmental safety.

Robotic systems that meet these criteria and follow the guidelines should be prioritized for commercialization and large-scale implementation. These guidelines not only provide a roadmap for developing autonomous and sustainable mechanical weed management systems but also highlight future research directions aimed at tackling the global challenge of weed management. Advanced mechanical weeding approaches should focus on net yield as a critical performance indicator, optimizing system design to maximize crop yields for specific varieties. By aligning system performance with yield outcomes, mechanical weeding systems can become a transformative solution for precision agriculture.

## 5 Conclusion

The ongoing research and advancements in the integration of Artificial Intelligence, machine vision and robotics into mechanical weed control systems has advanced significantly, positioning intelligent mechanical weeding systems as an important context in precision agriculture sector. This review examines a broad body of literature synthesizing insights on mechanical weeding methods, sensing and detection technologies, integrated robotic platforms and actuation strategies that define the state of the art in autonomous weed management. The evidence shows that intelligent mechanical weeders leveraging high resolution RGB cameras, sensors and DL models such as YOLO and ResNet, are steadily improving in their capacity to identify and localize weeds under real field conditions. These systems, paired with adaptive end-effectors provide an increasingly reliable means of controlling inter and intra-row weeds with greater precision compared to traditional approaches.

Over the past two decades, intelligent mechanical weeding systems have progressed from conceptual prototypes to increasingly functional field-ready platforms, yet their widespread adoption remains constrained by persistent challenges. Accurate weed–crop differentiation under diverse field conditions is still difficult, as lighting variability, morphological similarities, and occlusion often reduce classification accuracy and compromise real-time actuation. Synchronizing detection models with mechanical tool actuation in real time also requires high computational resources, creating trade-offs between speed and accuracy when deployed on embedded or edge devices. Crop injury risks, inconsistent performance across soil types and terrains, and high system costs further limit confidence among farmers and stakeholders. These obstacles highlight that the next generation of intelligent mechanical weeding systems must be not only technologically advanced but also economically viable, scalable, and easy to use.

Promising research directions are emerging to address these gaps. Lightweight and efficient deep learning algorithms optimized for edge computing platforms will be essential to achieve accurate and real-time detection in resource-constrained environments. Adaptive end-effectors capable of operating across different crop systems, weed growth stages, and soil conditions can minimize crop disturbance while improving robustness. Likewise, reliable navigation will increasingly depend on sensor fusion approaches that combine LiDAR, GPS, IMUs, and computer vision to deliver stable performance in heterogeneous and unstructured terrains. Modular platform designs with intuitive user interfaces will lower adoption barriers by enabling farmers to operate and troubleshoot systems without specialized expertise. Just as important, more long-term and large-scale field trials must be conducted under varying agro-ecological conditions to validate performance, ensure reliability, and strengthen confidence in these technologies.

The future of intelligent weeding will likely rest on hybrid approaches that integrate mechanical control with complementary methods such as selective see-and-spray systems or laser-based techniques. Such integration can enhance precision, reduce herbicide reliance, and provide a more holistic and sustainable weed management solution. By combining the mechanical precision of robotics with the flexibility of other innovative methods, these hybrid platforms can address both environmental sustainability and economic feasibility. In conclusion, while significant challenges remain, the convergence of AI-driven detection, adaptive mechanical tools, and advanced robotic platforms is paving the way for a new era of weed management. Intelligent mechanical weeders hold the potential to reduce chemical inputs, improve crop yields, and promote sustainable farming practices. As research continues to refine detection accuracy, actuation efficiency, navigation reliability, and system affordability, these technologies are poised to become indispensable components of precision agriculture, offering practical and scalable solutions to the pressing challenge of sustainable food production.
